# Combination of the Endogenous *lhcsr1* Promoter and Codon Usage Optimization Boosts Protein Expression in the Moss *Physcomitrella patens*

**DOI:** 10.3389/fpls.2017.01842

**Published:** 2017-10-31

**Authors:** Manuel Hiss, Lucas Schneider, Christopher Grosche, Melanie A. Barth, Christina Neu, Aikaterini Symeonidi, Kristian K. Ullrich, Pierre-François Perroud, Mareike Schallenberg-Rüdinger, Stefan A. Rensing

**Affiliations:** ^1^Plant Cell Biology, Faculty of Biology, University of Marburg, Marburg, Germany; ^2^BIOSS Centre for Biological Signaling Studies, University of Freiburg, Freiburg im Breisgau, Germany

**Keywords:** *Physcomitrella patens*, codon usage, chlorophyll a/b binding protein, promoter, codon bias, green fluorescent protein (GFP), *lhcsr1*, fluorescence normalization

## Abstract

The moss *Physcomitrella patens* is used both as an evo-devo model and biotechnological production system for metabolites and pharmaceuticals. Strong *in vivo* expression of genes of interest is important for production of recombinant proteins, e.g., selectable markers, fluorescent proteins, or enzymes. In this regard, the choice of the promoter sequence as well as codon usage optimization are two important inside factors to consider in order to obtain optimum protein accumulation level. To reliably quantify fluorescence, we transfected protoplasts with promoter:GFP fusion constructs and measured fluorescence intensity of living protoplasts in a plate reader system. We used the red fluorescent protein mCherry under 2x *35S* promoter control as second reporter to normalize for different transfection efficiencies. We derived a novel endogenous promoter and compared deletion variants with exogenous promoters. We used different codon-adapted green fluorescent protein (GFP) genes to evaluate the influence of promoter choice and codon optimization on protein accumulation in *P. patens*, and show that the promoter of the gene of *P. patens* chlorophyll a/b binding protein *lhcsr1* drives expression of GFP in protoplasts significantly (more than twofold) better than the commonly used 2x *35S* promoter or the rice *actin1* promoter. We identified a shortened 677 bp version of the *lhcsr1* promoter that retains full activity in protoplasts. The codon optimized GFP yields significantly (more than twofold) stronger fluorescence signals and thus demonstrates that adjusting codon usage in *P. patens* can increase expression strength. In combination, new promotor and codon optimized GFP conveyed sixfold increased fluorescence signal.

## Introduction

The strength of protein expression can be influenced by many factors including outside factors such as culture conditions, or inside factors as codon usage or transcription/translation system (Ullrich et al., 2015). Both promoter sequence and coding sequence can be optimized to improve final protein accumulation. The constructs of the first stable mutant lines in *Physcomitrella patens* contained resistance cassettes controlled by the cauliflower mosaic virus (CaMV) *19S* and *35S* promoters and the *Rhizobium radobacter* (previously *Agrobacterium tumefaciens*) nopaline synthase gene (*nos*) promoter (Schaefer et al., 1991). As *P. patens* was further developed as a plant model, classical strong angiosperm promoters such as the *Oryza sativa* (rice) *actin1* promoter (McElroy et al., 1990) or the *Zea mays* (maize) *ubiquitin* promoter (Christensen et al., 1992) were used successfully to drive protein accumulation in moss (Bezanilla et al., 2003; Horstmann et al., 2004). Inducible expression systems have also been established in *P. patens*, such as the beta-estradiol inducible one (Kubo et al., 2013) or the induction by elevated temperature using a *Glycine max* (soybean) heat shock protein promoter (Saidi et al., 2005). In a previous study, the activity of several promoters was studied by transient *P. patens* protoplast transfection of promoter:luciferase fusion constructs; here, the *actin1* promoter showed 10 times the expression level of the single CaMV *35S* promoter and 1.6 times the level of the 2x CaMV *35S* promoter (Horstmann et al., 2004). The same study also included endogenous 5′ sequences of the genes α1,3-fucosyltransferase and β1,2-xylosyltransferase (*fuc-t, xyl-t*) that were further characterized *via* deletion constructs, with the 5′*-fuc-t* showing almost double activity as compared to the single CaMV *35S* promoter. To confer strong expression, other endogenous promoters were used, e.g., different *tubulin* (Jost et al., 2005) or *actin* promoters (Weise et al., 2006), all of which showed stronger expression than the CaMV *35S* promoter, that was later shown to yield only mediocre expression in *P. patens*, especially in the dark (Saidi et al., 2009).

Chlorophyll a/b binding (CAB) proteins are part of the light harvesting complex (LHC) of photosynthetic eukaryotes. *Cab* promoter sequences have been used as strong endogenous and exogenous promoters, e.g., in the charophyte alga *Closterium peracerosum–strigosum–littorale* complex, where an endogenous promoter of a *cab* gene has been used to drive expression of fluorescence marker genes (Abe et al., 2008). The rice *cab1R* gene promoter was used for transient expression of β-glucuronidase (*gus*) in *Nicotiana tabacum*, *Z. mays* and *O. sativa* leaves (Luan and Bogorad, 1992). In addition to the common LHC gene set, a LHC-like protein called LHCSR (or Li818) is present in association with LHC in phylogenetically diverse algae as *Chlamydomonas reinhardtii* (Li et al., 2000) and *Ectocarpus siliculosus*, but not in seed plants (Koziol et al., 2007; Dittami et al., 2010). LHC-like protein expression is regulated by light and stress conditions. In *P. patens* two *lhcsr* gene copies have been identified (Gerotto et al., 2011). *lhcsr1* is induced by high light (450 μmol/m^-2^ s^-2^), whereas *lhcsr2* is expressed in low temperature and low light conditions (Gerotto et al., 2011). Together with the protein PSBS, the LHCSR proteins are responsible for the non-photochemical quenching in *P. patens* (Alboresi et al., 2010). The mechanisms of photoprotection of *lhcsr1 via* two dissipative states has recently been revealed (Kondo et al., 2017), as well as its modulation *via* zeaxanthin binding and low pH (Pinnola et al., 2017).

A second important inside factor influencing protein production is the codon usage in the RNA sequence (Quax et al., 2015). Due to the redundancy of the genetic triplet code, almost all amino acids are encoded by more than one codon. Which codon is used to which degree depends, e.g., on the species and on availability of tRNAs (Komar, 2016). Codon usage can be influenced by mutations and affects expression speed or accuracy. When trying to express a gene sequence from one species in another species, the codon usage often needs to be adjusted to fit the target species’ codon frequencies. For example, the codon usage of the GFP, that is used in many organisms, e.g., to localize proteins by tagging, has been optimized for different organisms like *Saccharomyces cerevisiae* (yeast) (Cormack et al., 1997) or the alga *C. reinhardtii* (Fuhrmann et al., 1999), leading to stronger GFP signals. For plants a soluble modified GFP (smGFP) was created by site-directed mutagenesis that shows stronger GFP accumulation in *Arabidopsis thaliana* and therefore a stronger signal than the wild-type GFP (Castillo-Davis et al., 2002). Codon usage can vary not only between species but also within a species. In a subgroup of genes from one species, a bias for certain codons can be found, e.g., in highly expressed genes of *Caenorhabditis elegans*, *Drosophila melanogaster*, and *A. thaliana* codon usage differs from more weakly expressed genes of the same species (Duret and Mouchiroud, 1999). In *P. patens*, this codon usage bias seems to be driven by a combination of weak natural selection and the predominant mutational biases (Szovenyi et al., 2017).

Here we used the endogenous promoter sequence of *lhcsr1* to drive gene expression of the GFP in *P. patens* and compared it to the double CaMV *35S* (2x p*35S*) and the rice *actin1* promoter (McElroy et al., 1990). In parallel, we designed two GFP versions with different codon usage and evaluated their GFP accumulation to the smGFP using a novel bimodal fluorescence readout system that allows to normalize the signal of interest in order to account for fluctuations in transfection efficiency. We find that the *lhcsr1* gene promoter increases signal intensity 1.7-fold as compared to the 2x *35S* promoter. In combination with the codon-optimized GFP the signal even increases 5.7-fold as compared to the 2x *35S* promoter.

## Results

### Fluorescence Readout System

**FIGURE 1:**
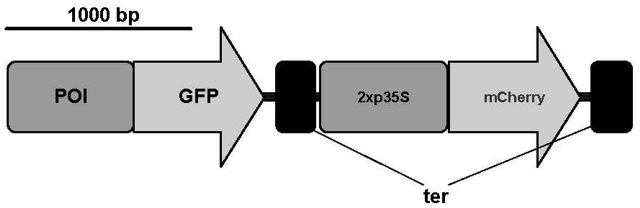
Constructs used for transient expression of GFP and mCherry. Promoter of interest (POI); green fluorescent protein sequence (GFP); 2x *35S* promoter (2x p*35S*); *35S* terminator (ter). Scale bar represents 1,000 base pairs. A modified pAM-PAT vector (accession number: AY436765) was used as backbone for transient constructs. Figure was prepared with IBS (Liu et al., 2015).

We designed a novel bimodal readout system that can be used to measure reporter protein fluorescence *in vivo*. We opted to use a microplate reader to measure fluorescence to allow high throughput measurement. For all fluorescence measurements, we used living protoplasts in transfection regeneration medium (Hohe et al., 2004). Initial tests showed that a minimum number of 2,000 protoplasts are necessary to get a signal above background fluorescence (Supplementary Figure 1). The constructs used for transient transfection contained the promoter:GFP fusion and additionally a normalization cassette consisting of the 2x *35S* promoter, the mCherry gene sequence and a *35S* terminator (**Figure 1**). We selected the 2x *35S* promoter because it is widely used in the *P. patens* community and shows a medium gene expression in *P. patens*, which makes it a good candidate for comparisons with new promoters. The fluorescence from this second reporter was used to normalize the GFP fluorescence values, which show high between-experiment variation due to different transformation efficiencies (Supplementary Figure 2 and Supplementary Table 1). By integrating 2xp*35S*:mCherry into the same plasmid as the GFP fusion of interest, we also account for uptake of multiple plasmids during transformation. To test the feasibility of our plate reader measurement system, we performed protoplastation of protonemal tissue from stable mutant lines that express GFP or mCherry (Perroud et al., 2011), mixed the protoplasts and found that we can measure both signals in parallel from the same well. Additionally, we prepared a dilution series and found a linear relationship between number of protoplasts and fluorescence intensity for both reporters (Supplementary Figure 3).

### Highly Expressed Genes Show Codon Usage Bias in *P. patens*

**FIGURE 2:**
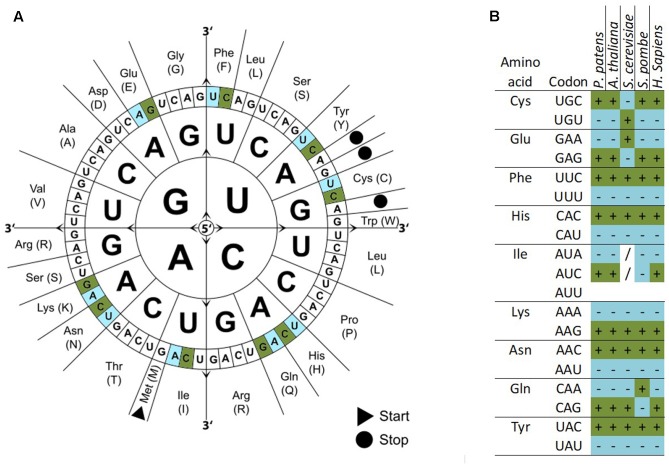
Codon usage bias in *P. patens* as compared to other selected organisms. Codon usage differs between all genes and highly expressed genes. Codons significantly over represented in highly expressed genes are colored dark green/marked by a (+) and significantly under represented codons are colored light blue/marked by a (–) (*p*-adjusted < 0.05, Fisher’s exact test). **(A)** Codon sun adapted from Wikimedia Commons with biased codons marked in green (more abundant) or blue (less abundant). **(B)** Table of amino acids, codons, and codon bias in different species. Codons without bias are marked (/).

We calculated the codon frequencies for all v1.6 genes of *P. patens* and grouped them by expression strength based on a broad range of transcriptome microarray experiments (Hiss et al., 2014). The top 233 highly expressed genes (0.9%) are enriched in the Gene Ontology (GO) terms translation, gene expression, and protein synthesis (Supplementary Figure 4). By comparing the codon usage of these highly expressed genes with the codon usage of all genes, we find a significant codon usage bias (Fisher’s Exact Test, FDR adjusted *p* < 0.05) for nine amino acids, namely Glu, Phe, Tyr, Cys, His, Gln, Ile, Asn, and Lys (**Figure 2A** and Supplementary File 1). These codons are preferred not only in *P. patens* highly expressed genes but also in other organisms [**Figure 2B**; *Arabidospsis thaliana* (Wright et al., 2004; Morton and Wright, 2007), *Saccharomyces cerevisiae* (dos Reis and Wernisch, 2009), *Schizosaccharomyces pombe* (Hiraoka et al., 2009), and *Homo sapiens* (van Hemert and Berkhout, 1995)]. Using microarray data (Hiss et al., 2014), we can confidently detect the first biased codon usage (Lys) already within the top 1,967 highly expressed genes (7.3% of all genes measured by the array), and the codon usage bias for all nine amino acids can be detected based on the top 291 highly expressed genes (1.1%, Supplementary File 2). Thus, by using the biased codon usage as an indicator, we detect around 7% of the *P. patens* genes as highly expressed. These genes also show high expression in microarray-based transcriptome studies [Supplementary Figure 5, (Hiss et al., 2014; Ortiz-Ramirez et al., 2015)]. For the highly expressed genes, we detect a GC bias toward higher GC content and a lower effective number of codons (ENC) as compared to the overall gene set (Supplementary Figure 6).

To determine whether the codon bias we have seen in highly expressed genes in *P. patens* can be used to enhance protein expression, we prepared GFP variants with adapted codon usage. Starting from the smGFP (Davis and Vierstra, 1998), we changed all triplets either to the one preferred in highly expressed genes, or to the one not preferred. This led to 39 changes in the coding sequence for “GFPhigh” and 58 changes for “GFPlow,” respectively (Supplementary Figure 7 and Supplementary Table 2). The GC content of the smGFP was 43.7% as compared to 49.1% (GFPhigh) and 35.6% (GFPlow). In both adapted GFP versions, the resulting amino acid sequence was not changed.

All three GFP versions were combined with different promoters 2xp*35S*, p*actin1*, and p*lhcsr1* (1,956 bp upstream region of the *Physcomitrella patens lhcsr1* gene) and the mCherry normalization cassette. These constructs were transfected transiently into *P. patens* protoplasts and the fluorescence signal of GFP and mCherry measured *in vivo* with a microplate reader system.

**FIGURE 3:**
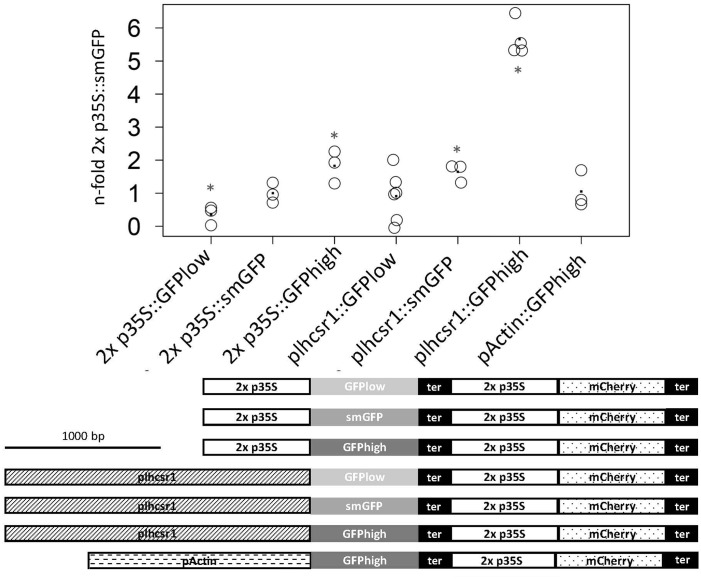
Measurement of normalized GFP signal of seven different promoter:GFP fusion constructs. GFP and mCherry signal was measured with a microplate reader in transiently transfected protoplasts. GFP signal intensity is normalized by mCherry signal intensity and the 2x p*35S*:smGFP was set to one. Average value is marked by a dot (3–6 replicates). Asterisks mark significant difference from 2x p*35S*:smGFP (^∗^*p* < 0.05, Student’s *t*-test). The seven fusion constructs are depicted below the graph. Scale bar represents 1,000 base pairs. 2x *35S* promoter (2x p*35S*), *lhcsr1* promoter (p*lhcsr1*), rice actin 1 promoter (p*Actin*), soluble modified GFP (smGFP), codon adapted GFP (GFPlow, GFPhigh), and *35S* terminator (ter).

The measurements were normalized and the 2x *35S* promoter in combination with the smGFP signal set to one. The rice *actin1* promoter:GFPhigh fusion is at 1.0 ± 0.6-fold in our system and therefore not significantly different from the 2x *35S* promoter with the same GFP version (*p* = 0.15, Student’s *t*-test). We see a shift in signal intensity for the 2x *35S* promoter constructs with different GFP versions, with the GFPhigh giving the strongest signal at 1.8 ± 0.5-fold and the GFPlow the weakest at 0.4 ± 0.3-fold. Different signal intensities can also be seen with the different p*lhcsr1*:GFP combinations starting with GFPlow at 0.9 ± 0.8-fold to smGFP at 1.7 ± 0.3-fold up to GFPhigh at 5.7 ± 0.4-fold (**Figure 3**).

### *lhcsr1* Promoter Shows Strong Expression

**FIGURE 4:**
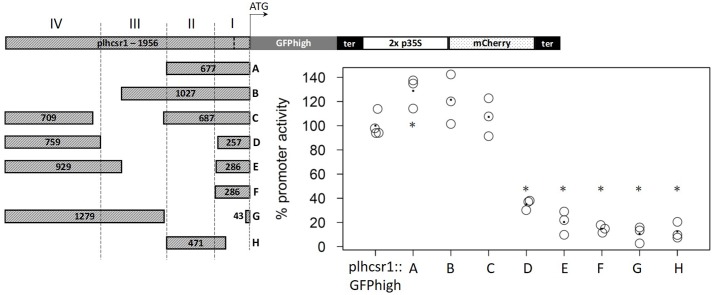
Comparison of percentage promoter activity of nine different promoter fragments in GFPhigh fusion constructs. GFPhigh and mCherry signal was measured in transiently transfected protoplasts. GFPhigh signal intensity is normalized by mCherry signal intensity and the full length p*lhcsr1* was set to 100%. Average value is marked by a dot (3–4 independent transfection experiments). Asterisks mark significant difference from the full length p*lhcsr1* (^∗^*p* < 0.05, Student’s *t*-test). Left: p*lhcsr1* promoter fragments (A–H) with sizes in base pairs. Regions I–IV based on restriction enzyme sites are marked by dashed lines. *lhcsr1* gene promoter (p*lhcsr1*), codon adapted GFP variant (GFPhigh), *35S* terminator (ter), and 2x *35S* promoter (2x p*35S*). The transcription start site predicted by the Plant Promoter Database (Hieno et al., 2014) is marked in the full length p*lhcsr1* promoter with a dashed line.

We selected a strong and constitutively expressed gene from our transcriptome microarray data which cover several developmental stages and perturbations (Hiss et al., 2014). To find a suitable endogenous promoter we calculated the coefficient of variation (c. v.) of the expression values of all genes represented on the Combimatrix microarray (Wolf et al., 2010) and selected genes with high expression values and low coefficient of variation, leading to candidate genes with a high expression across many developmental stages and perturbations. Additionally we filtered for genes whose 3 kbp upstream region does not overlap with another gene or transposable element (TE). The 3 kbp gene-free upstream region was chosen in order to prevent the regulatory sequences of the gene of interest to fall into the sequence of another gene, and in order not to select atypically short genes (Zimmer et al., 2013). The 3kbp upstream region further should not contain TEs because they are known to be silenced *via* methylation of the corresponding genomic region (Zemach et al., 2010; Widiez et al., 2014). We chose the cab protein LHCSR1 (Phypa_169593, Pp1s213_80V6.1, Pp3c9_3440V3.1) since the promoters of *cab* genes of plants have successfully been used as strong endogenous and exogenous promoters before (Abe et al., 2008). We used about 2,000 bp upstream of the coding sequence (Chr09:1,975,316…1,977,272) as putative promoter for cloning and subsequent transfection (expression profile of the *lhcsr1* gene, see Supplementary Figure 8). The *lhcsr1* gene does not show the bias in codon usage we generally observe in the top highly expressed genes (Supplementary Table 3). The constructs containing the full p*lhcsr1* promoter show higher signals than the corresponding 2x p*35S* constructs (**Figure 3**). We prepared eight shortened promoter sequences by restriction digestion of the original 1,956 bp sequence (**Figure 4**, constructs A–H). All promoter versions were fused to GFPhigh:*35S*-terminator and the mCherry normalization cassette, transfected transiently into moss protoplasts and the GFP and mCherry signal measured *in vivo* with the microplate reader system.

Based on restriction enzyme sites, we divided the 1,956 bp promoter sequence into four regions (**Figure 4**). Region I contains the 202 bp 5′-UTR and 84 bp of the upstream region. Region II consists of a 391-bp fragment, Region III of a 520-bp fragment, and Region IV of the 759 bp at the 5′ end of the chosen promoter sequence. The removal of the regions IV and III without modifying the rest of the promoter does not strongly affect promoter activity, and the remaining fragments display an activity between 121 and 129% compared to the full length (1,956 bp) promoter (**Figure 4**, constructs A, B, and C). None of the other partial deletions abolish totally the promoter activity, most of the regions I and II deletions display 12–20% total p*lhcsr1* value (**Figure 4**, constructs D–H). The only exception is the construct D that does not contain the activating region II, but still retains 35% promoter activity (**Figure 4**, construct D). The region IV appears to contain a domain able to activate transcription once it is fused to the 5′-UTR. This is especially interesting in contrast to construct E which has longer fragments for regions I and IV but a lower signal intensity.

**FIGURE 5:**
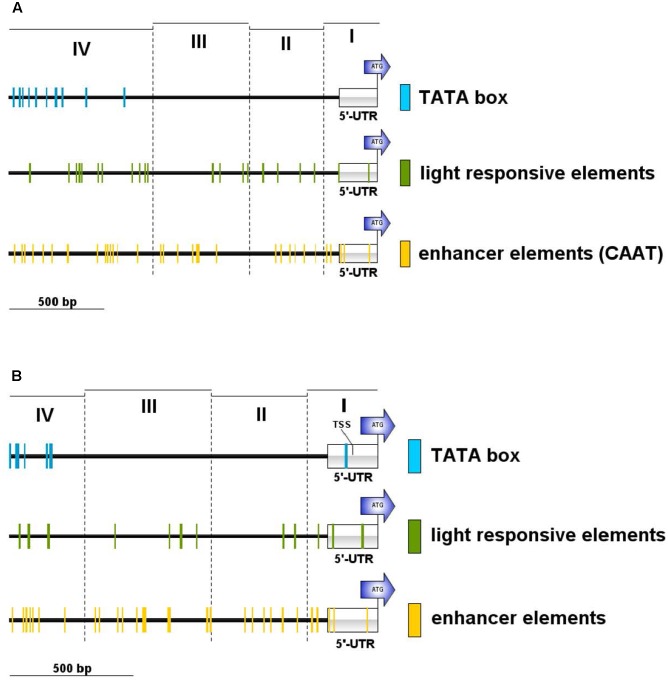
Motif occurrences in the *P. patens lhcsr1* promoter. The promoter sequence was screened against the plant *cis*-acting regulatory DNA elements database PLACE **(A)** and the plant *cis*-acting regulatory element database PlantCARE **(B)**. The 1,956 bp upstream region of the *lhcsr1* gene was split into four regions based restriction enzyme sites (*cf*. **Figure 4**). The four regions are marked by dashed lines. Colored lines mark occurrence of light responsive elements (green), TATA box motifs (blue), and enhancer element motifs (yellow). Motifs situated on the plus and minus strand are shown. Position of the 5′ untranslated region (5′-UTR) and the start codon of the *lhcsr1* gene (ATG) are shown. PlantCARE database only used the first 1.5 kbp of the promoter sequence. Regions I and II need to be present to achieve full promoter activity.

**FIGURE 6:**
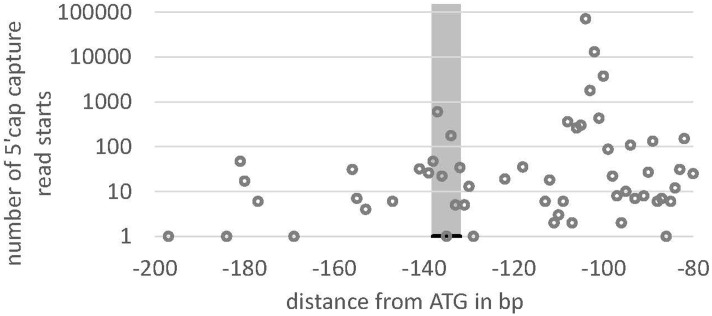
Read starting position from 5′ Cap-capture sequencing data for *lhcsr1*. Number of reads (*y*-axis) of RNA-Seq data from *P. patens* gametophores that start within the 5′-UTR at the position –80 to –200 bp upstream of the translation start (ATG) of the *lhcsr1* gene. Gray bar marks TATA box predicted by PlantCARE. Note the peaks at –100 and at the TATA box, potentially representing two transcription start sites (TSS). The upstream TSS at –100 is the one present in the Plant Promoter Database (*cf*. **Figure 4**).

We searched the potential promoter sequence for motifs using Signal Scan and the plant *cis*-acting regulatory DNA elements database [PLACE, (Higo et al., 1999)] as well as the Plant *cis*-acting regulatory element (PlantCARE) database. PLACE finds several known *cis*-acting elements that are associated with light-regulated genes, e.g., GT1-sites (Green et al., 1988) or I-boxes (Giuliano et al., 1988), whereas many TATA boxes are found in region IV, further away from the ATG but not in the 5′-UTR or the regions II and III (**Figure 5A**). PlantCARE predicts a TATA box core promoter element for both *lhcsr* genes within the 5′-UTR at around -130 bp of the translation start (**Figure 5B**). The transcription start site (TSS) data implemented in the Plant Promoter database (Hieno et al., 2014) support this TATA box based on 5′ end sequencing of the *lhcsr1* gene with a TSS found 102 bases upstream of the start codon (Supplementary Figure 9). This TATA box is further supported by a possible TSS present in 5′ Cap-capture sequencing data available at the *P. patens* CoGe representation (**Figure 6**).

## Discussion

Promoter:reporter gene constructs can be used to compare expression strength between different promoters. The signal can be read out after cell lysis (Horstmann et al., 2004) or directly from living cells (Thevenin et al., 2012). The readout system established in this study measures the fluorescence signal of transiently transfected protoplasts expressing the reporter genes GFP and mCherry. We use a comparatively low-priced multi-well, multi-wavelength plate reader to measure the fluorescence signal of living protoplasts, while a recent study used a FACS system for measurements to investigate transcription factor activities in living cells (Thevenin et al., 2012). Since transformation efficiencies vary a lot between different transformations and to account for the uptake of varying numbers of plasmids into one cell (Supplementary Figure 2), we introduced a mCherry normalization cassette into all of our constructs. This novel readout system reduces the variation we see in our measurements and thereby helps to differentiate between different promoter strength. This system also allows to operate on lower numbers of protoplasts and biological replicates. We assume that detectable fluorescence correlated in linear fashion with protein amount, an assumption supported by the measurement of protoplasts of stable expressing lines (Supplementary Figure 3).

Codon usage of *P. patens* has been calculated and published based on expressed sequence tag (EST) data that were created before the genome sequence was published (Rensing et al., 2005; Stenoien, 2005) and recently based on the gene models predicted from genome sequencing (Szovenyi et al., 2017). In our analysis of codon usage bias based on v1.6 gene models in *P. patens*, we detect a bias in highly expressed genes to prefer certain codons. The same preferences can be seen in other organisms (**Figure 2B**). We can detect a bias for the first codons already within the top 7.3% expressed genes (Supplementary File 1). These highly expressed genes can be confirmed using microarray data (Supplementary Figure 5), and show a bias toward higher GC content and lower ENC (Supplementary Figure 6). In *C. reinhardtii*, codon usage, rather than GC content, was found as key determinant of gene expression efficiency. However, the nucleotide composition was found to feed back to the chromatin state of the promoter region (Barahimipour et al., 2015). To show that the detected codon bias, acting on translation, can be used for biotechnological applications, we created two GFP versions with adapted codon usage but identical amino acid sequence. Starting from the soluble-modified GFP version that gives high signals in particle bombardment transformation assays in *P. patens* (Cho et al., 1999), we exchanged codons to create a GFP that contains codons biased toward high protein expression (GFP high) and a GFP version that contains the opposite codons (GFP low). With the GFP version using preferred codons, we measure higher GFP signals, similar to studies in maize using optimal human codons (Chiu et al., 1996). The effect of our codon changes on protein expression can already be measured when using the 2x *35S* promoter that gives medium expression strength in *P. patens*, and is more pronounced under the control of the stronger *lhcsr1* promoter (**Figure 3**).

Since the 2x *35S* promoter is not the best choice for strong gene expression in biotechnological protein production in *P. patens*, we selected a candidate promoter based on microarray analysis covering important tissues, several stress conditions, and hormone treatments. This includes typical production conditions like protonemal tissue and protoplasts in light. We selected the promoter of *lhcsr1*, a gene coding for a cab protein. LHCSR1 and LHCSR2 are both part of the light harvesting complex and are activated by light. Even though they are mainly expressed during conditions with photosynthetically active tissue, we see a medium RNA expression level under dark conditions in our microarray data (Supplementary Figure 8). This medium expression in darkness should allow the use of this promoter in selection cassettes where diurnal expression is preferred to allow for the upkeep of resistance. In contrast, the single *35S* promoter was found to be inactive in darkness (Saidi et al., 2009). Compared to the 2x *35S* and the *actin1* promoter we see a 2- to 6-fold higher expression when using the *lhcsr1* promoter, based on transcriptional activation. In our expression and readout system, the 2x *35S* and *actin1* promoters do not show significant difference in expression strength, contrary to previous reports in which the *actin1* promoter reached 1.6 times the expression level of the 2x *35S* promoter in a firefly luciferase reporter system (Horstmann et al., 2004). This difference could be due to the different normalization method that uses a second, separate plasmid as transfection control. This resulted in higher variation of the relative luciferase activity between different transfections with a median of coefficients of variation at 0.42 (Supplementary Table 4). Our GFP reporter system with the normalization cassette included into the same plasmid as the promoter of interest provides less variance with the median of coefficients of variation at 0.22 (Supplementary Table 5).

As putative *lhcsr1* promoter sequence, we selected the 1,956 bp upstream of the ATG of the *lhcsr1* gene. Since biotechnological applications will benefit from a shorter promoter sequence, we created shortened versions of the original sequence. We divided the original fragment into four regions based on restriction sites and were able to identify a promoter fragment of 677 bp that shows full promoter activity. Shorter fragments and fragments that lack the region II (**Figure 4**) show a weaker promoter activity. Removing the 5′-UTR from the 677 bp fragment also results in a low promoter activity, suggesting a regulatory role for the 5′-UTR. Analyzing the promoter fragments for binding sites, PlantCARE detects a TATA box in the 5′-UTR as well as sites known to enhance RNA expression and several binding sites for light regulated transcription factors. With our fully active 677-bp promoter fragment showing at least twofold expression strength of the 2x *35S* promoter, we provide a useful alternative for strong protein expression in *P. patens*. In combination with our codon optimized GFPhigh the 2x *35S* promoter shows a twofold expression strength increase as compared to the 2x *35S* promoter with non-optimized GFP. However, the combination of *lhcsr1* promotor and GFPhigh led to the highest observed activity with up to sixfold expression rate relative to the before mentioned standard.

Our study shows that a significant increase in protein production can be achieved by using suitable combinations of promoter and codon optimization, tackling transcription as well as translation efficiency. In contrast, combining even a strong promoter with genes not codon-optimized for high expression leads to low expression (**Figure 3**). These findings will be especially helpful in biotechnological and proteomics applications producing proteins in the moss *P. patens*, but also to drive, e.g., fluorescence tags and selectable markers. For these future applications, the generation of stable mutant lines with the expression cassettes integrated into the genome will be necessary to test whether the strong expression can also be seen in the genomic context. This will also allow to evaluate the expression strength in different tissues and conditions.

## Materials and Methods

### Plant Material

*Physcomitrella patens* Gransden (Rensing et al., 2008) was cultivated on solidified [1% (w/v) agar] mineral medium, also known as modified (Reski and Abel, 1985) Knop’s (1868) medium, on 9-cm petri dishes enclosed by laboratory film at 22°C with a 16-h-light/8-h-dark regime under 70 μmol m^-2^ s^-1^ white light (long-day conditions).

### Codon Usage

To assess codon usage bias, the coding sequences of v1.2 gene models on the Combimatrix microarray experiments from Hiss et al. (2014) that show expression values above the detection limit were analyzed (26,856 genes). Genes were sorted according to their expression level and genes with a normalized fluorescence intensity above 200,000 (567 genes, 2.1%) were termed strongly expressed genes whereas a value of 450,000 (233 genes, 0.87%) put them into the group of highly expressed genes. The codon frequencies for each group were calculated with *R* and afterward a Fisher’s Exact Test was used for each of the 64 codons to find significant changes in codon frequencies between the groups. To account for the high number of statistical tests, multiple testing adjusted *p*-values were calculated with the *R* function *p*.adjust and the significance level was set at adjusted *p* < 0.05.

The assessment at which expression level a codon usage bias can be detected was based on the coding sequences of v1.6 gene models with data based on the v1.2 Combimatrix microarray experiments. Custom software used to calculate codon usage and adjusted *p*-values can be found in the Github repository https://github.com/kullrich/bio-scripts/tree/master/codonusage.

### Generation of Transient Constructs

Plasmids for transient transfection were assembled by Golden Gate Cloning using the *Sap*I enzyme. A modified pAM-PAT vector (accession number: AY436765) was used for cloning of 2x p*35S* constructs. The multiple cloning site (MCS) was removed, and together with a chloramphenicol resistance gene and a ccdb kill cassette, two *Sap*I restriction sites were introduced to create the vector pAM-PAT-*Sap*I. To accommodate the insertion of other promoters, the pAM-PAT-*Sap*I vector was modified by restriction with *Xho*I and *Sal*I, removing the 2x *35S* promoter. For cloning reactions promoter and reporter sequences were amplified with primers containing the *Sap*I restriction site with matching overhang. *Sap*I-cut vector, amplified promoter and amplified reporter were incubated with *Sap*I and T4 ligase in ligase buffer and after 1 h incubation at room temperature transformed into TOP10 *E. coli* cells. The mCherry gene was amplified from a modified p123 vector (kindly provided by Michael Bölker) with *Sap*I restriction sites as overhang (mCherry-SapI_fwd, mCherry-SapI_rev) and inserted into the pAM-PAT vector behind the 2x p*35S* sequence. The 2xp*35S*:mCherry fusion was amplified with *Not*I restriction sites as overhang (*Not*I-p35SS_fwd, *Not*I-mCherry) and ligated into the pAM-PAT vector *via* the *Not*I site. Although insertion was not directed by specific restriction sites the clones obtained always contained the 2x p*35S*:mCherry in the same direction as the promoter:GFP insert. To test whether a read-through from the *lhcsr1* promoter could lead to increased expression of mCherry, we removed the 2x *35S* promoter in front of the mCherry. We could not detect a mCherry signal above background when using these constructs (Supplementary Table 6). Plasmid DNA was extracted by the Bibdo protocol (Birnboim and Doly, 1979) or with the NucleoBond Xtra Midi Kit (Macherey-Nagel, Germany).

Modified GFP versions were synthetized by Geneart (Germany) and delivered in the pMA-T vector, inserted *via* the *Sfi*I restriction site. The GFP versions were amplified with primers containing *Sap*I restriction sites as overhang (GFP_high-for_SAP, GFP_high_rev, GFP_low_for, GFP_high_rev). During primer design, the second codon of the smGFP was changed from serine (S) to valine (V). To test whether this has an effect on expression, we compared the GFP/mCherry ratio of the 2x p*35S*:S-V_smGFP to a correct 2x p*35S*:smGFP. The plate reader measurements did not show a difference between the S and V versions of the smGFP (Supplementary Figure 10). 1,956 bp of genomic sequence upstream of the coding sequence for the cab protein (Phypa_169593) were amplified by PCR with primers containing the *Sap*I restriction site as overhang (p169593_for, p169593_rev). Shortened versions of the *lhcsr1* gene promoter sequence were created by restriction digest and blunt/compatible end ligation with *Eco*RV + *Sal*I (A), *Eco*RV + *Bcl*I (B), *Dra*II (C), StuI (D), *Bcl*I + *Bgl*II (E), *Eco*RV + *Bgl*II (F), and *Sal*I + *Xho*I (G). The *Eco*RV + *Sal*I construct without 5′-UTR (H) was amplified from the full length construct with *Sap*I restriction sites as overhang (sh_pCAB_for, sh_pCAB_rev). The rice *actin1* (McElroy et al., 1990) promoter sequence was amplified by PCR from the PIG-AN vector (Schallenberg-Rudinger et al., 2017) with primers containing the *Sap*I restriction site as overhang (pActin_Sap_for, pActin_Sap_rev).

Cloning success was tested by selection on ampicillin and either by colony PCR or test digestion. Positive candidate plasmids were sent for Sanger sequencing to GATC (Konstanz, Germany) or Macrogen (Amsterdam, Netherlands). The primers used for plasmid construction are shown in Supplementary Table 6.

### Moss Protoplast Transfection

Transfection protocol was adapted from (Hohe et al., 2004). Regularly disrupted protonemal tissue in a 200 mL liquid culture, pH 5.8 was adjusted to 60 mg/L dry weight and transferred to 200 mL liquid Knop medium pH 4.5. After 5–6 days, the culture was harvested by sieving (100 μm sieve). Protonemal tissue was equilibrated in 12 mL 0.51M Mannitol (pH 5.6–5.8) for 30 min, 4 mL Driselase solution (4%) was added and incubated for 1–2 h on a slowly tumbling shaker. The protoplast solution was sieved first on a 100 μm, then on a 50 μm sieve and afterward centrifuged 10 min at 50 *g*. Supernatant was removed, protoplasts resuspended in 10 mL 0.51 M Mannitol and centrifuged 10 min at 50 *g*. Supernatant was removed, protoplasts resuspended in 10 mL 0.51 M Mannitol and protoplast number counted on a Fuchs-Rosenthal counting chamber. Protoplast suspension was centrifuged for 10 min at 50 *g*, supernatant removed and a concentration of 1.2 × 10^6^ protoplasts per mL adjusted with MMM medium [MMM medium, 0.51 M Mannitol, 15 mM MgCl_2_, 0.1% w/v 2-(N-morpholino)ethanesulfonic acid, pH 5.6]. For transfection, 100 μL DNA in 0.1 M Ca(NO_3_)_2_, 250 μL protoplast suspension, and 350 μL PEG solution (40% PEG 400 in MMM medium) were mixed during a 30 min incubation time. To slowly dilute the transfection solution, first 1 mL of MMM medium is added and mixed, next 2, 3, 4, and 5 mL are added and mixed every 5 min. Suspension was centrifuged for 10 min at 50 *g* and protoplasts resuspended in regeneration medium (0.28 M glucose and 0.24 M mannitol in Knop medium, pH 5.8). For transient transfections, circular plasmid was used. DNA amounts used for transient transfections were 10–50 μg. After transfection, protoplasts were left to regenerate in 1 mL of regeneration medium.

### Plate Reader Measurements

Fluorescence intensity measurements were performed in a FLUOstar microplate reader (BMG Labtech, Germany) with transfected *P. patens* protoplasts. Sample volumes of 100 μL (up to 30,000 protoplasts) were placed into black 96-well microplates with transparent bottom (Greiner Bio-one, Austria). The samples were detected using the bottom optic, orbital averaging with 2 mm diameter and 15 flashes per well. For GFP and mCherry fluorescence, emission filters at 485 and 584 nm as well as excitation filters at 520 and 620 nm were used with 10 nm bandpass width. Regeneration medium was used as the blank value and non-transfected protoplasts as background control. Blank values were subtracted and the ratio of GFP and mCherry signals was calculated for each well to normalize for the transfection efficiency. Measurements were done 6–7 days after transfection since time course experiments found the highest fluorescence signal intensity after this time period (Supplementary Figure 11).

### Gene Ontology (GO) Analyses and Visualization

The GO bias analyses used Fisher’s Exact Test to calculate *p*-values. Multiple testing corrected (Benjamini and Hochberg, 1995) *q*-values were calculated in *R* with the function *p*.adjust (R Development Core Team, 2008). Word cloud visualizations were created using the online tool wordle^1^. Word size is proportional to the -log10(*q*-value) and over-represented GO terms were colored dark green if *q* ≤ 0.0001 and light green if *q* > 0.0001. Under-represented GO terms were colored dark red if *q* ≤ 0.0001 and light red if *q* > 0.0001.

## Author Contributions

CG, CN, LS, MB, and MH prepared the constructs, transfected protoplasts, and analyzed data. AS and KU calculated codon frequencies. SR conceived of the work. MS-R, P-FP, and SR supervised the project. MH, MS-R, P-FP, and SR designed the experiments. MH and SR wrote the manuscript with contributions by all authors.

## Conflict of Interest Statement

The authors declare that the research was conducted in the absence of any commercial or financial relationships that could be construed as a potential conflict of interest.

## Footnotes

1 http://www.wordle.net/
