# Supplementary material for: Combination of the Endogenous lhcsr1 Promoter and Codon Usage Optimization Boosts Protein Expression in the Moss Physcomitrella patens
Source: Front Plant Sci. 2017 Oct 31;8:1842. doi: 10.3389/fpls.2017.01842 (PMC5671511; doi:10.3389/fpls.2017.01842)
Supplement: Supplementary file 2 [file Table_1.docx]

Supplementary Material

Combination of the endogenous lhcsr1 promoter and codon usage optimization boosts protein expression in the moss *Physcomitrella patens*

Manuel Hiss^1^, Lucas Schneider^1,2^, Christopher Grosche^1^, Melanie A. Barth^1^, Christina Neu^1^, Aikaterini Symeonidi^1,3^, Kristian K. Ullrich^1,4^, Pierre-François Perroud^1^, Mareike Schallenberg-Rüdinger^1,5^, Stefan A. Rensing^1,6*^

^1^ Plant Cell Biology, Faculty of Biology, University of Marburg, Marburg, Germany

^6^ BIOSS Centre for Biological Signaling Studies, University of Freiburg, Germany

**Correspondence:**

Stefan A. Rensing

Tel. +49 6421 28-21940

Fax +49 6421 28-22190

stefan.rensing@biologie.uni-marburg.de

@RensingStefan

^2^ Present address: Institute for Transfusion Medicine and Immunohematology, Johann-Wolfgang-Goethe University and German Red Cross Blood Service, Frankfurt am Main, Germany

^3^ Present address: Institute for Research in Biomedicine (IRB Barcelona), Barcelona, Spain

^4^ Present address: Max Planck Institute for Evolutionary Biology, Ploen, Germany

^5^ Present address: IZMB–Institut für Zelluläre und Molekulare Botanik, Abteilung Molekulare Evolution, Universität Bonn, Bonn, Germany

**Supplemental Figure 1: Plate reader fluorescence measurements of a dilution series of living Physcomitrella patens wild type (WT) and mutant (GFP) protoplasts**

Fluorescence intensity of different protoplast numbers of wild type and a stable GFP was measured as well as empty wells and regeneration medium. X-axis shows number of protoplasts and y-axis shows fluorescence values of the plate reader measurement. Error bars show standard deviation of three technical replicates. Asterisks mark significant (p < 0.05) differences between GFP and the WT as well as between GFP and regeneration medium.

**Supplemental Figure 2: Plate reader fluorescence measurements of living Physcomitrella patens wild type (WT) and mutant (GFP) protoplasts**

Average fluorescent measurement raw data of protoplasts from multiple transfections with regeneration medium, transiently transfected wild type protoplasts and protoplasts from a stable GFP line as controls. Four different promoter:GFP constructs were used for transfection. Error bars show standard deviation from 4 - 6 transfections.

**Supplemental Figure 3: Plate reader fluorescence measurements of living Physcomitrella patens mutant protoplasts**

Protoplasts of stable GFP or mCherry mutants were mixed in one well and GFP as well as mCherry fluorescence intensity was measured. X-axis shows number of protoplasts of each strain and y-axis shows background corrected fluorescence values of the plate reader measurement. Error bars show standard deviation of three technical replicates. Linear regression for both dilution series was calculated and R^2^ values are given.

**Supplemental Figure 4: Gene Ontology word cloud of biological process terms of the 233 highly expressed genes of Physcomitrella patens**

Expression values from microarray experiments covering the major life stages and several perturbations (Hiss et al. 2014). Under represented categories are shown in red, over represented categories in green, word size proportional to –log(p-value) from Fisher’s exact test (for details see Methods).

**Supplemental Figure 5: Boxplots of normalized mean fluorescence values from Combimatrix (A) and Nimblegen (B) microarray experiments of Physcomitrella patens**

Mean expression values from microarray experiments of *Physcomitrella patens tissues* from A: (Hiss, et al. 2014) and B: (Ortiz-Ramirez et al. 2015). “All genes” shows normalized mean expression values of all genes on the Combimatrix microarray (A) or the Nimblegen microarray (B). “Subset” shows array-based normalized mean expression values of the top 7.3% genes described in this manuscript as highly expressed.

**Supplemental Figure 6: Boxplots of GC content (%) and effective number of codons (ENC) calculated from Physcomitrella patens v1.6 gene models**

“Subset” shows GC % and ENC of the top 7.3% highly expressed genes described in this manuscript; “background genes” shows GC % and ENC of the other 92.7% of genes.


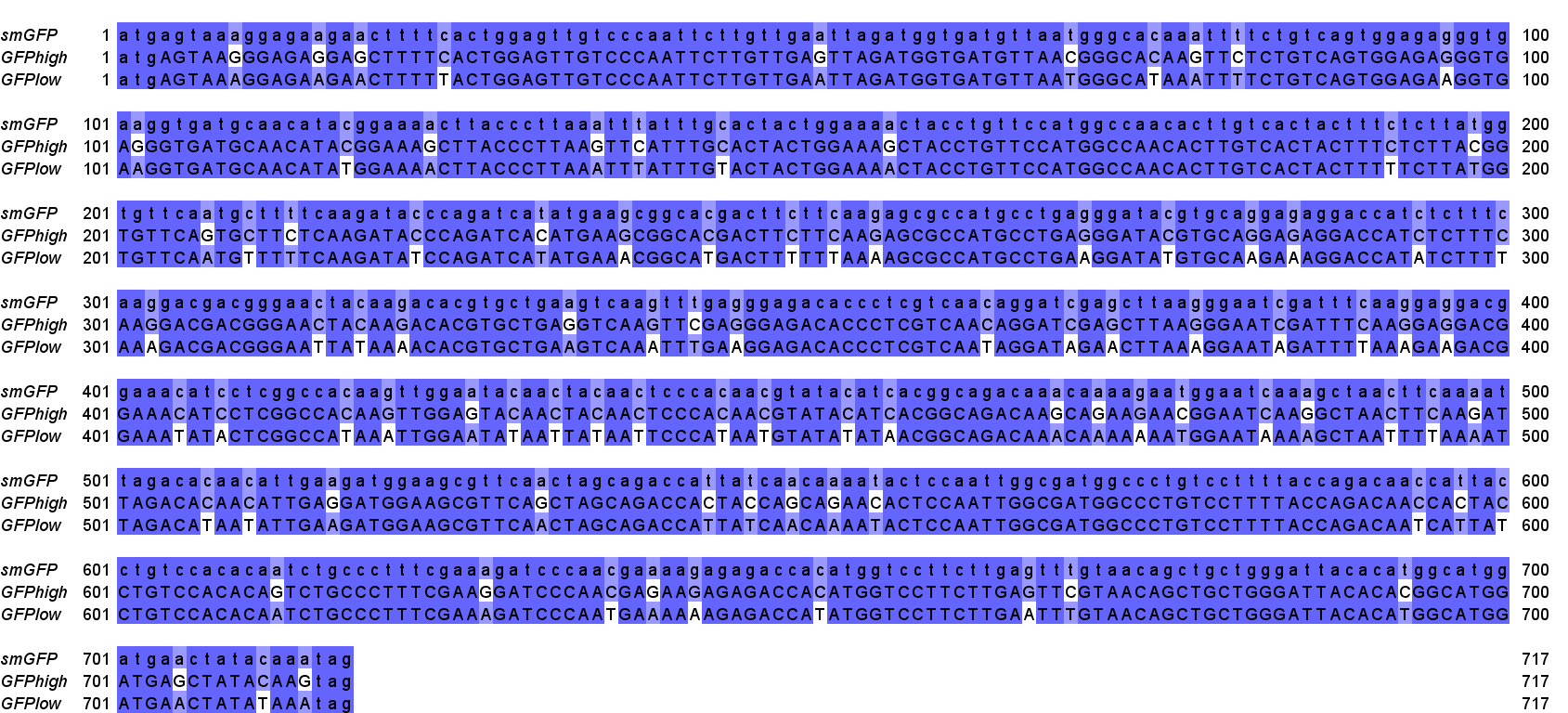


**Supplemental Figure 7: ClustalOmega nucleotide alignment of three GFP versions**

Starting from the soluble modified GFP (smGFP) several codons were changed to create a GFP version featuring codons biased towards high expression levels (GFPhigh) and one version adapted for low expression levels (GFPlow). Nucleotide changes are marked by white background. The amino acid sequence is not changed by these nucleotide changes.

A

B

**Supplemental Figure 8: Expression profile of the genes lhcsr1 (Phypa_169593, Pp3c9_3440V1.1) and lhcsr2 (Phypa_233731, Pp3c15_11070V3.1).**

From microarray (A) and RNA-Seq experiments (B). Y-axis shows normalized fluorescence units (A) or RPKM values (B) with error bars showing the standard deviation of 2-3 biological replicates.


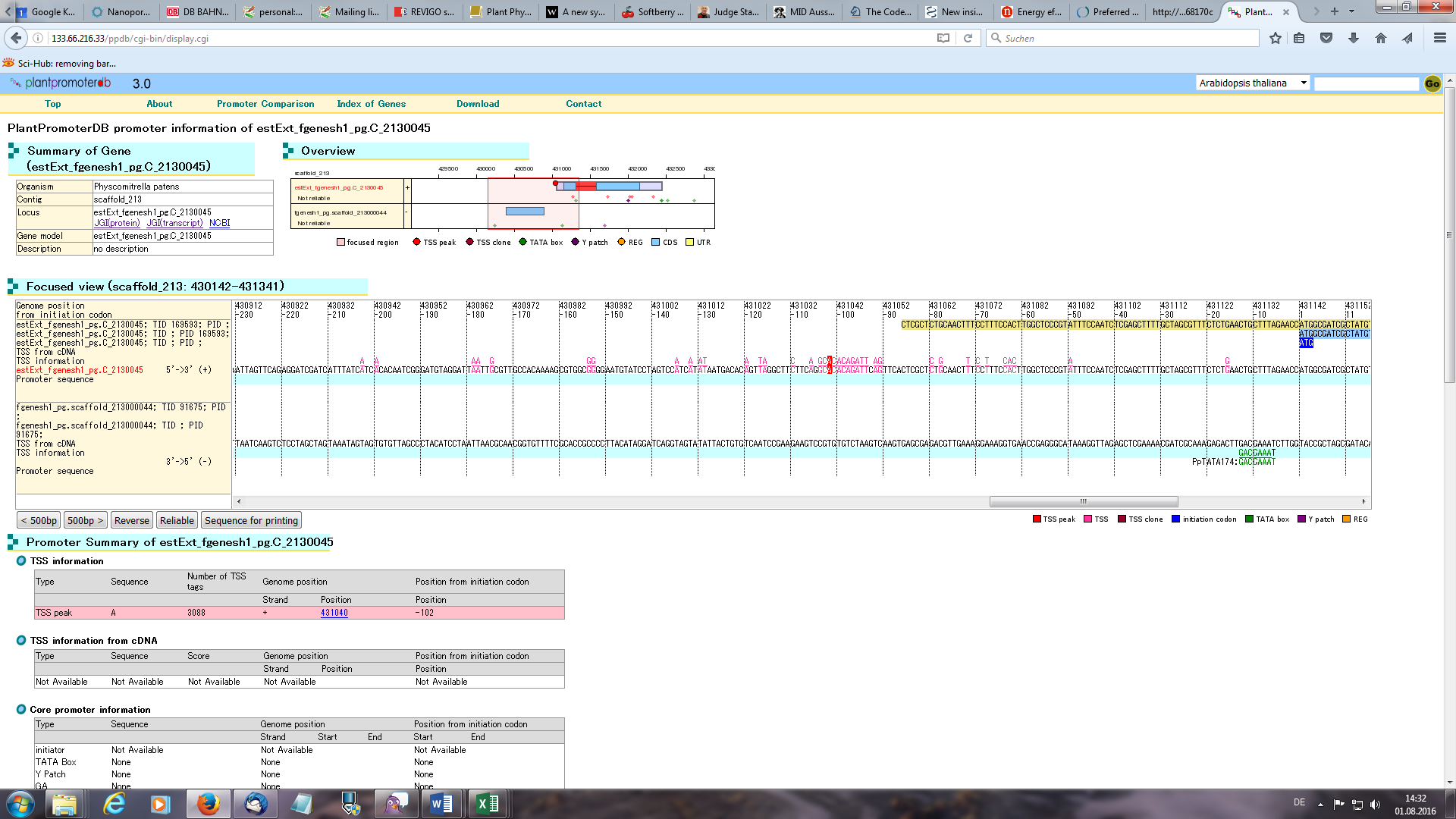


**Supplemental Figure 9: Screenshot from the PlantPromoter DB for LHCSR1 (estExt_fgenesh1_pg.C_2130045).**

**Supplemental Figure 10: GFP/mCherry ratios from two promoter:GFP constructs**

The 2x 35S:smGFP contains Serine at position 2 of the original smGFP, whereas the 2x 35S:S-V_smGFP has a Valine at this position. Both constructs were transiently transfected into protoplasts of *Physcomitrella patens* and fluorescence measured *in vivo* in a plate reader.

**Supplemental Figure 11: Bar chart of average GFP fluorescence values during a time course of four to ten days after transfection**

*Physcomitrella patens* protoplasts were transfected with the double CaMV 35S promoter:GFPhigh fusion construct and left to recover in regeneration medium. Aliquots were taken daily and GFP fluorescence was measured in the plate reader. Y-axis shows background corrected arbitrary fluorescence units. Error bars show standard deviation of three technical replicates.

**Supplemental Table 1: Plate reader fluorescent measurement data of Physcomitrella patens transfected protoplasts.**

Fluorescent measurement raw data of protoplasts from six different transfections (Transf. 1-6) with regeneration medium, wild type protoplasts and protoplasts from a stable GFP line as controls. Four different promoter:GFP constructs were used for transfection. Mean, standard deviation (st. dev.) and coefficient of variation (c.v.) were calculated.

**Supplemental Table 2: List of codon changes in the adapted GFP versions**

Starting from the soluble modified GFP (smGFP) codons for nine amino acids were changed to create a GFP version producing high expression levels (GFPhigh) and a GFP version producing low expression levels (GFPlow). Number of codons changed to create the GFPhigh version are shown with light grey background, and number of codons changed to create GFPlow are shown with dark grey background.

**Supplemental Table 3: List of used codons in the lhcsr1 gene (Phypa_169593, Pp1s213_80V6.1, Pp3c9_3440V3.1) coding for the chlorophyll a/b binding protein LHCSR1**

Number of codons preferred in highly expressed genes are shown with light grey background, and number of codons less preferred in highly expressed genes are shown with dark grey background. Codon numbers calculated at http://www.bioinformatics.org/sms2/codon_usage.html.

**Supplemental Table 4: List of constructs from (Horstmann et al. 2004)**

Promoter strength in relation to the CaMV 35S (1x) (MV[%]), standard deviation (SD) and coefficient of variation (cv) are given.

 **Supplemental Table 5: List of promoter-GFP constructs used in this publication**

First column shows construct name, second column the alphabetical code used in Figure 4 (main text). Columns 3-8 show the calculated GFP/mCherry ratio measured in independent transfections of P.patens protoplasts with their average given in column 9. Standard deviation (stdev) and coefficient of variation (c.v.) are given in the last two columns.

**Supplemental Table 6: Primers used for construct generation and screening**

**Supplemental File S1: Excel spreadsheet with codon frequencies for the top 233 and all genes.**

**Supplemental File S2: PDF with codon usage frequency spectrum (30 pages).**

**Cited references**

Hiss M, Laule O, Meskauskiene RM, Arif MA, Decker EL, Erxleben A, Frank W, Hanke ST, Lang D, Martin A, et al. 2014. Large-scale gene expression profiling data for the model moss *Physcomitrella paten*s aid understanding of developmental progression, culture and stress conditions. Plant J. Aug;79:530-539. Epub 2014/06/04.

Horstmann V, Huether CM, Jost W, Reski R, Decker EL. 2004. Quantitative promoter analysis in Physcomitrella patens: a set of plant vectors activating gene expression within three orders of magnitude. BMC Biotechnol. Jul 07;4:13.

Ortiz-Ramirez C, Hernandez-Coronado M, Thamm A, Catarino B, Wang M, Dolan L, Feijo JA, Becker JD. 2015. A transcriptome atlas of *Physcomitrella patens* provides insights into the evolution and development of land plants. Mol Plant. Dec 11;9:205-220.
