# Supplementary material for: Combination of the Endogenous lhcsr1 Promoter and Codon Usage Optimization Boosts Protein Expression in the Moss Physcomitrella patens
Source: Front Plant Sci. 2017 Oct 31;8:1842. doi: 10.3389/fpls.2017.01842 (PMC5671511; doi:10.3389/fpls.2017.01842)
Supplement: Supplementary file 3 [file File_2.pdf]

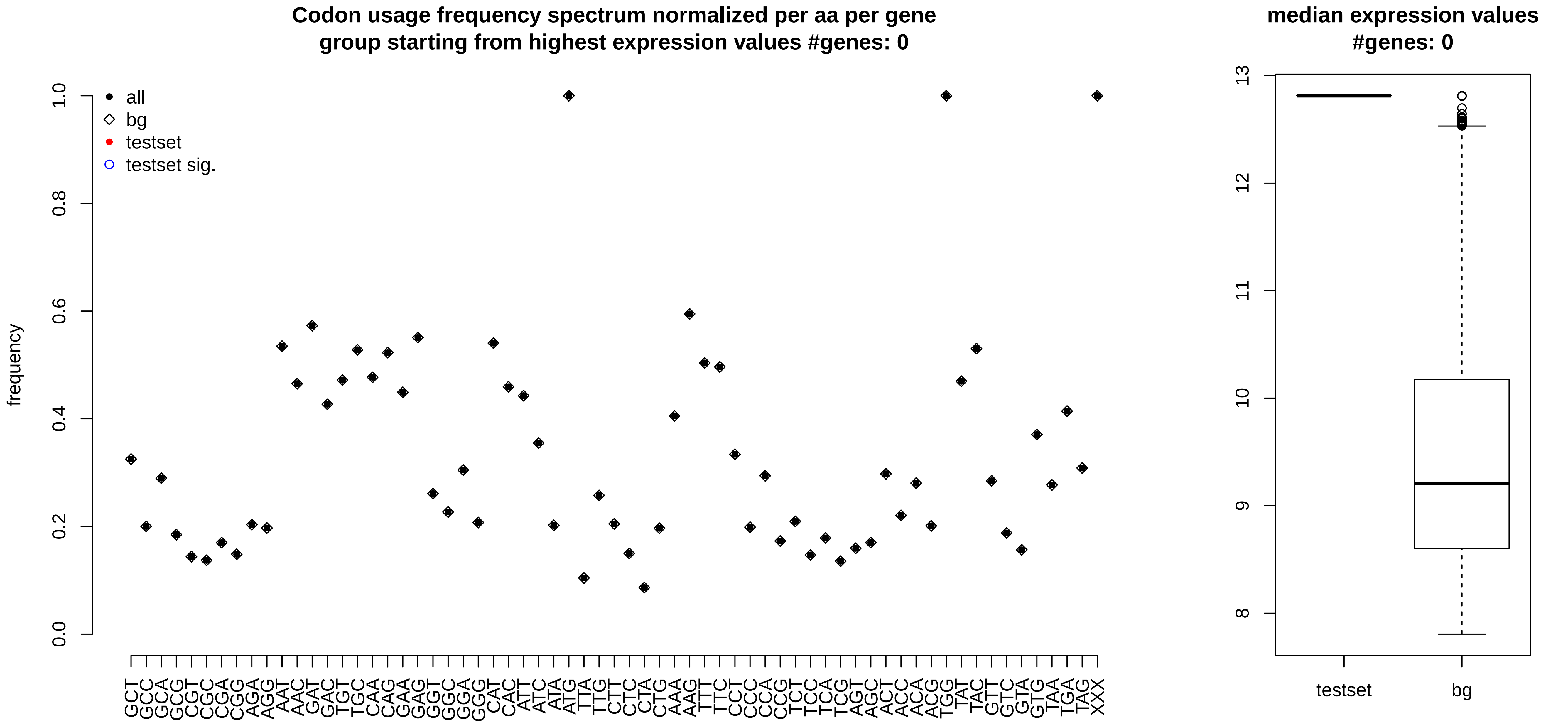

Codon usage frequency spectrum normalized per aa per gene
group starting from highest expression values #genes: 0
median expression values
#genes: 0
frequency
all
bg
testset
testset sig.
0.0
0.2
0.4
0.6
0.8
1.0
8
9
10
11
12
13
testset
bg
GCT GCC GCA GCG CGT CGC CGA CGG AGA AGG AAT AAC GAT GAC TGT TGC CAA CAG GAA GAG GGT GGC GGA GGG CAT CAC ATT ATC ATA ATG TTA TTG CTT CTC CTA CTG AAA AAG TTT TTC CCT CCC CCA CCG TCT TCC TCA TCG AGT AGC ACT ACC ACA ACG TGG TAT TAC GTT GTC GTA GTG TAA TGA TAG XXX

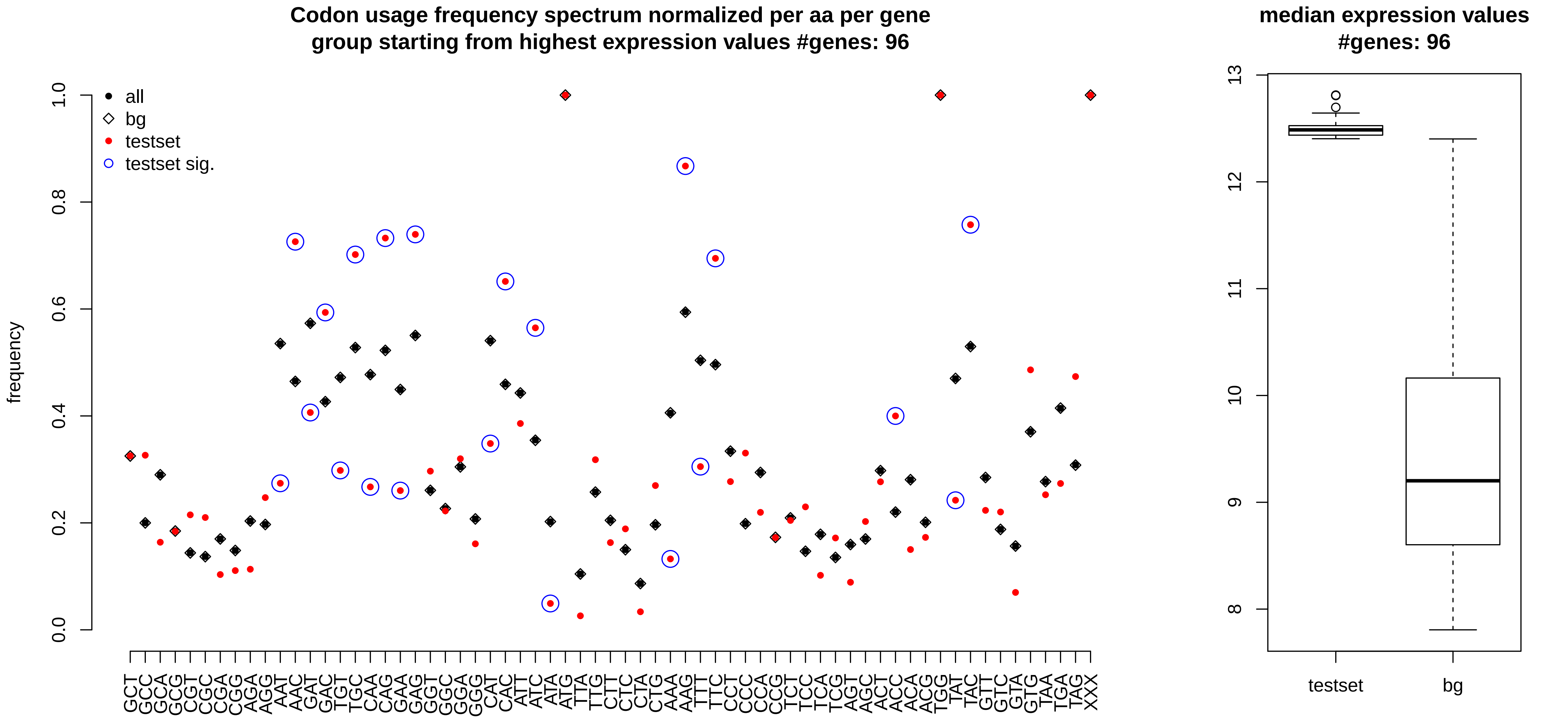

Codon usage frequency spectrum normalized per aa per gene group starting from highest expression values #genes: 96
frequency
all
bg
testset
testset sig.
0.0
0.2
0.4
0.6
0.8
1.0
GCT GCC GCA GCG CGT CGC CGA CGG AGA AGG AAT AAC GAT GAC TGT TGC CAA CAG GAA GAG GGT GGC GGA GGG CAT CAC ATT ATC ATA ATG TTA TTG CTT CTC CTA CTG AAA AAG TTT TTC CCT CCC CCA CCG TCT TCC TCA TCG AGT AGC ACT ACC ACA ACG TGG TAT TAC GTT GTC GTA GTG TAA TGA TAG XXX
median expression values #genes: 96
8
9
10
11
12
13
testset
bg

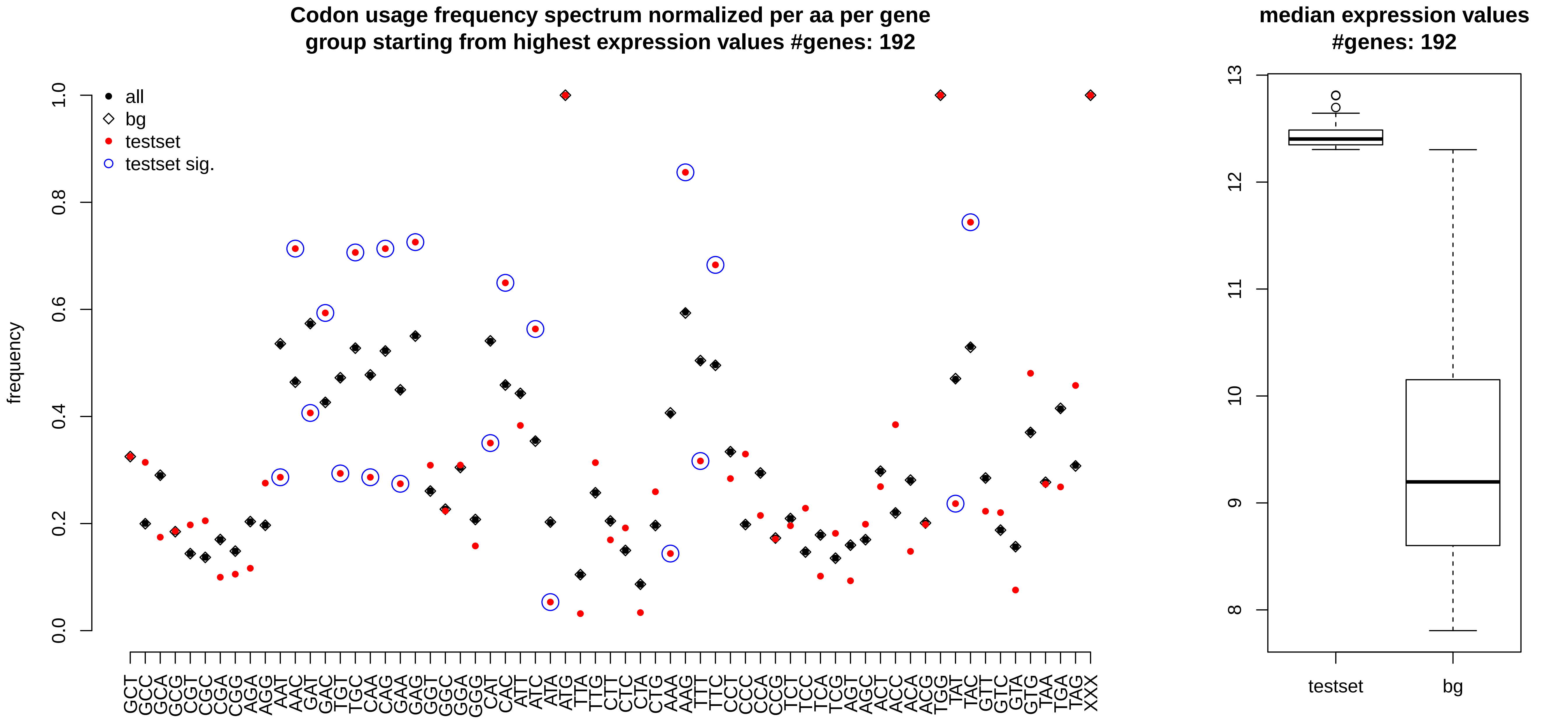

Codon usage frequency spectrum normalized per aa per gene
group starting from highest expression values #genes: 192
all
bg
testset
testset sig.
frequency
0.0
0.2
0.4
0.6
0.8
1.0
GCT GCC GCA GCG CGT CGC CGA CGG AGA AGG AAT AAC GAT GAC TGT TGC CAA CAG GAA GAG GGT GGC GGA GGG CAT CAC ATT ATC ATA ATG TTA TTG CTT CTC CTA CTG AAA AAG TTT TTC CCT CCC CCA CCG TCT TCC TCA TCG AGT AGC ACT ACC ACA ACG TGG TAT TAC GTT GTC GTA GTG TAA TGA TAG XXX
median expression values
#genes: 192
8
9
10
11
12
13
testset
bg

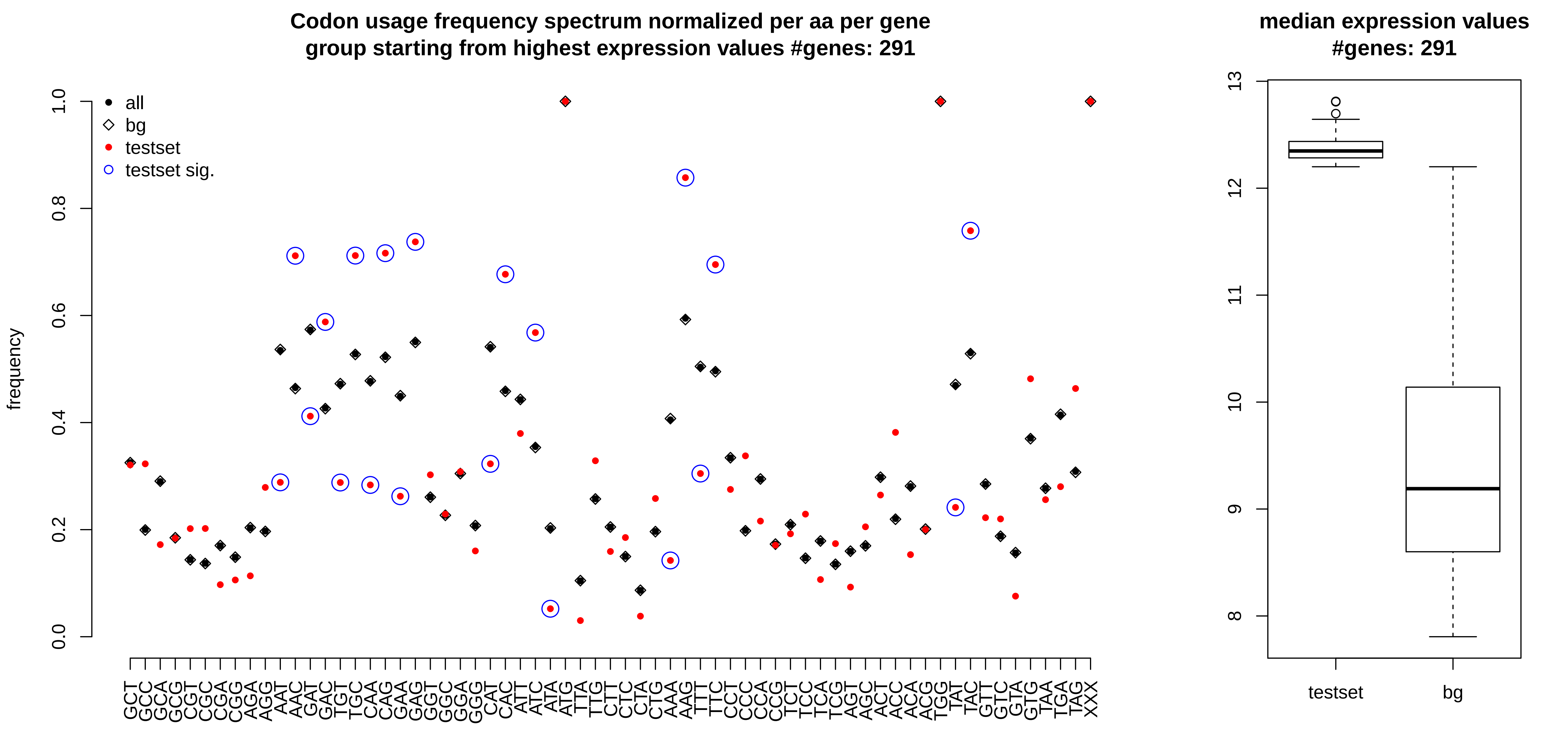

Codon usage frequency spectrum normalized per aa per gene group starting from highest expression values #genes: 291
frequency
all
bg
testset
testset sig.
0.0
0.2
0.4
0.6
0.8
1.0
GCT GCC GCA GCG CGT CGC CGA CGG AGA AGG AAT AAC GAT GAC TGT TGC CAA CAG GAA GAG GGT GGC GGA GGG CAT CAC ATT ATC ATA ATG TTA TTG CTT CTC CTA CTG AAA AAG TTT TTC CCT CCC CCA CCG TCT TCC TCA TCG AGT AGC ACT ACC ACA ACG TGG TAT TAC GTT GTC GTA GTG TAA TGA TAG XXX
median expression values #genes: 291
8
9
10
11
12
13
testset
bg

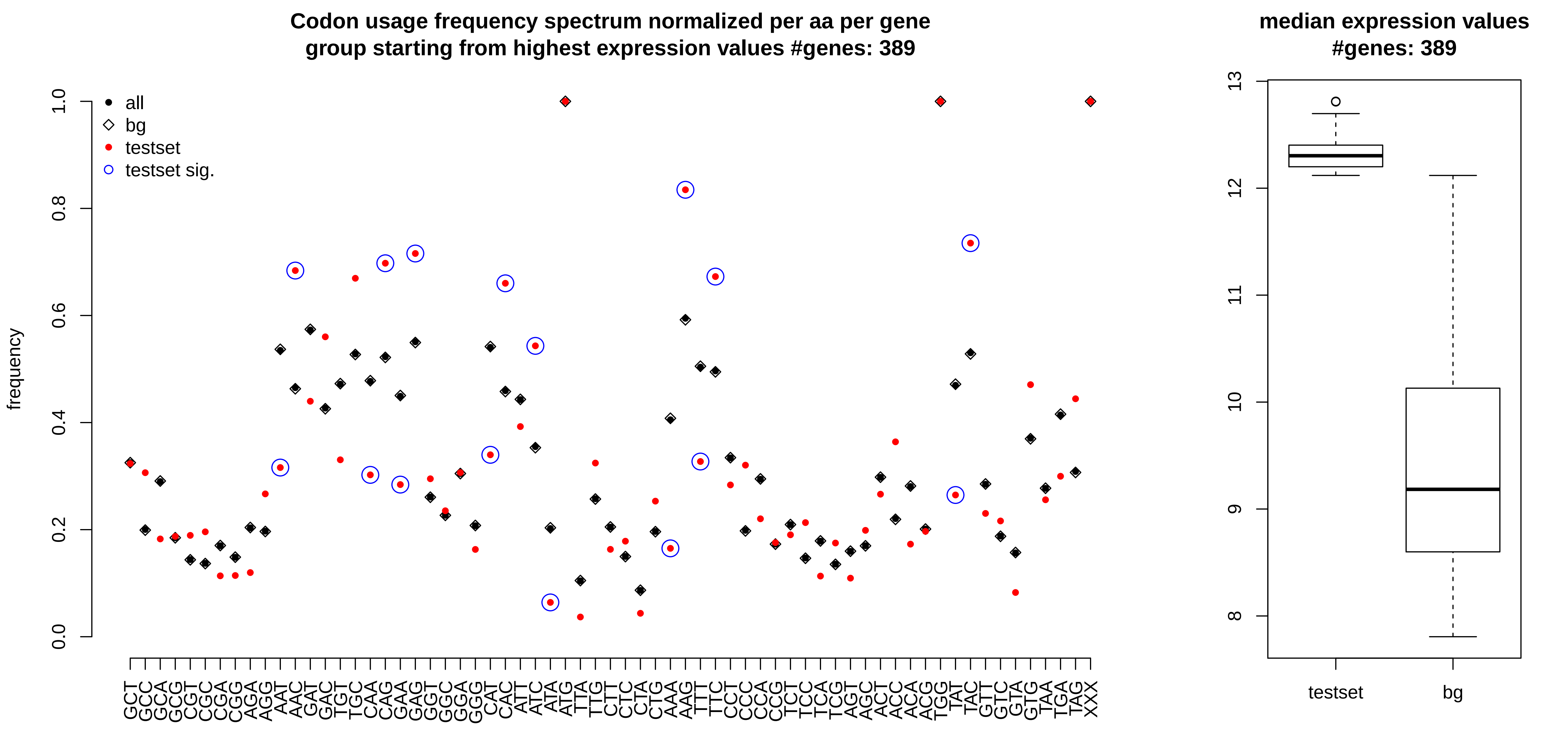

Codon usage frequency spectrum normalized per aa per gene
group starting from highest expression values #genes: 389
frequency
0.0
0.2
0.4
0.6
0.8
1.0
all
bg
testset
testset sig.
GCT GCC GCA GCG CGT CGC CGA CGG AGA AGG AAT AAC GAT GAC TGT TGC CAA CAG GAA GAG GGT GGC GGA GGG CAT CAC ATT ATC ATA ATG TTA TTG CTT CTC CTA CTG AAA AAG TTT TTC CCT CCC CCA CCG TCT TCC TCA TCG AGT AGC ACT ACC ACA ACG TGG TAT TAC GTT GTC GTA GTG TAA TGA TAG XXX
median expression values
#genes: 389
8
9
10
11
12
13
testset
bg

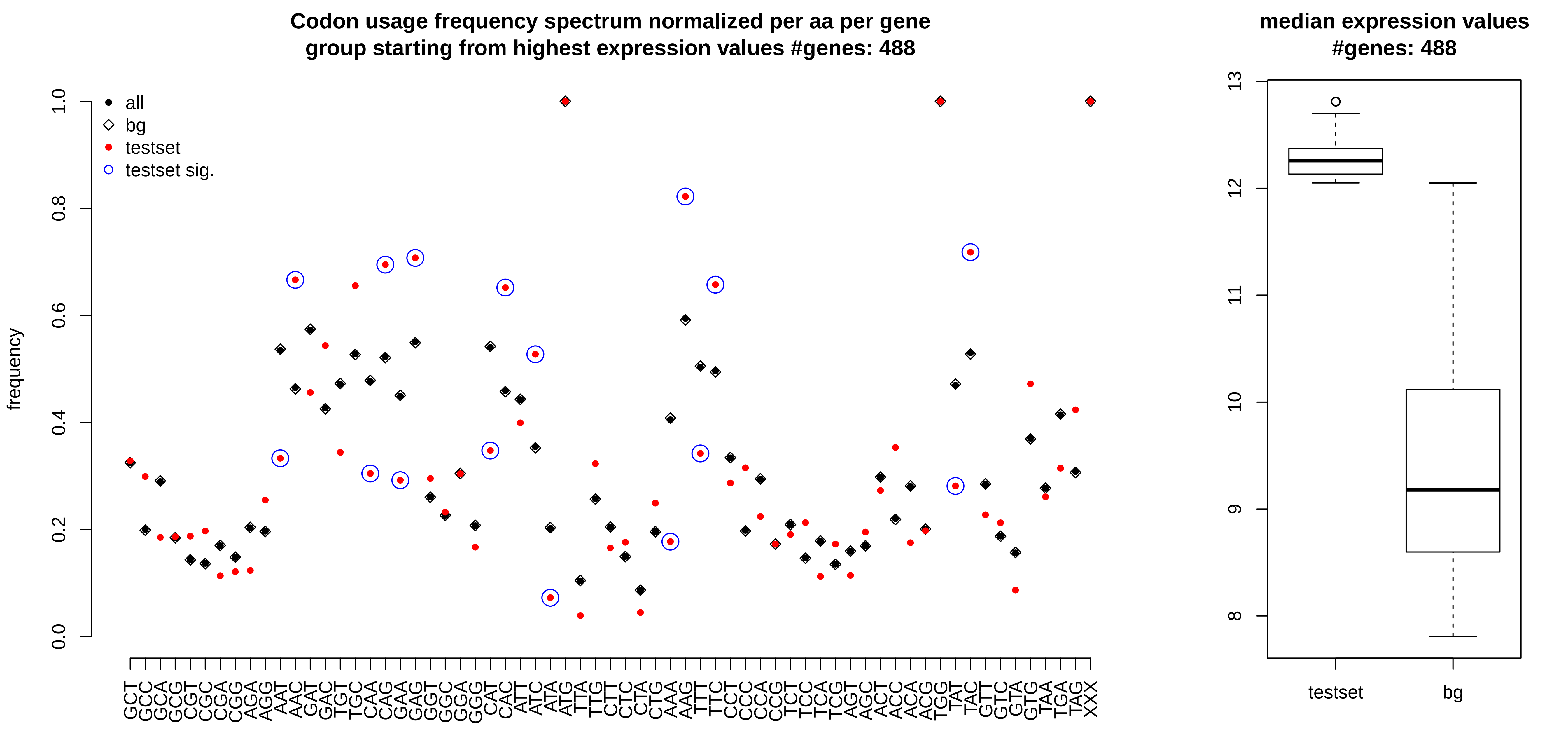

Codon usage frequency spectrum normalized per aa per gene
group starting from highest expression values #genes: 488
frequency
all
bg
testset
testset sig.
0.0
0.2
0.4
0.6
0.8
1.0
GCT GCC GCA GCG CGT CGC CGA CGG AGA AGG AAT AAC GAT GAC TGT TGC CAA CAG GAA GAG GGT GGC GGA GGG CAT CAC ATT ATC ATA ATG TTA TTG CTT CTC CTA CTG AAA AAG TTT TTC CCT CCC CCA CCG TCT TCC TCA TCG AGT AGC ACT ACC ACA ACG TGG TAT TAC GTT GTC GTA GTG TAA TGA TAG XXX
median expression values
#genes: 488
8
9
10
11
12
13
testset
bg

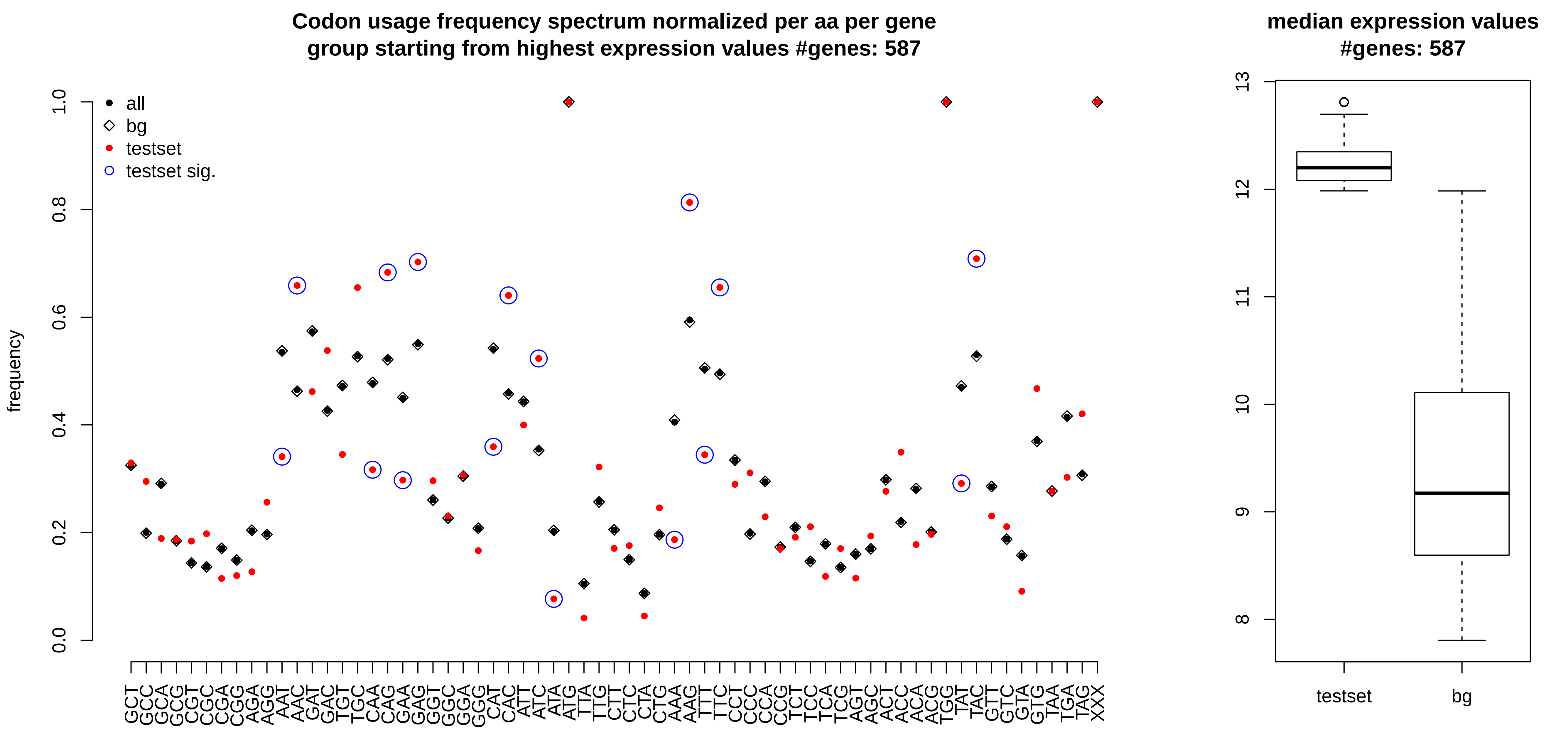

Codon usage frequency spectrum normalized per aa per gene
group starting from highest expression values #genes: 587
frequency
all
bg
testset
testset sig.
0.0
0.2
0.4
0.6
0.8
1.0
GCT GCC GCA GCG CGT CGC CGA CGG AGA AGG AAT AAC GAT GAC TGT TGC CAA CAG GAA GAG GGT GGC GGA GGG CAT CAC ATT ATC ATA ATG TTA TTG CTT CTC CTA CTG AAA AAG TTT TTC CCC CCA CCG TCT TCC TCA TCG AGT AGC ACT ACC ACA ACG TGG TAT TAC GTT GTC GTA GTG TAA TGA TAG XXX
median expression values
#genes: 587
8
9
10
11
12
13
testset
bg

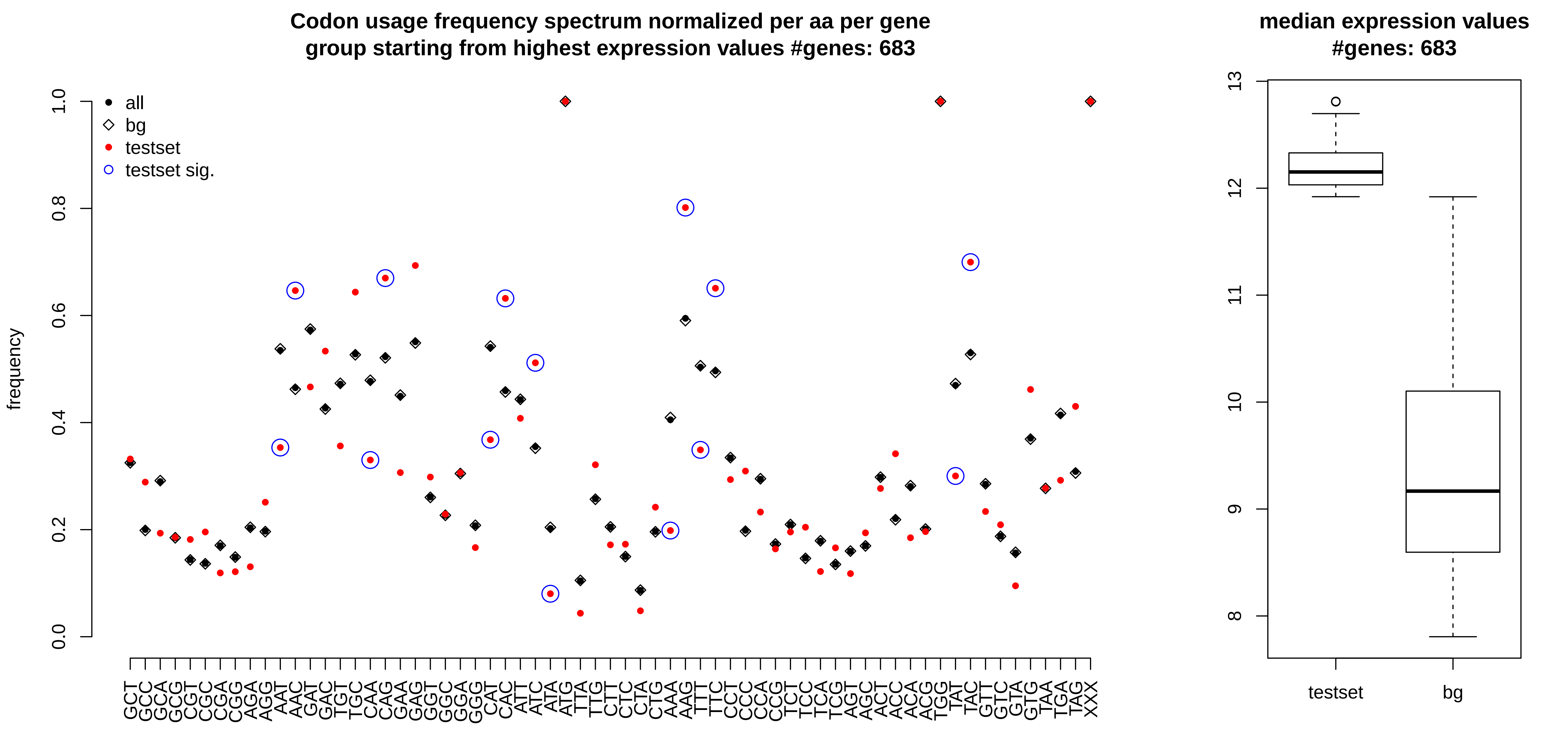

Codon usage frequency spectrum normalized per aa per gene
group starting from highest expression values #genes: 683
frequency
all
bg
testset
testset sig.
0.0
0.2
0.4
0.6
0.8
1.0
GCT GCC GCA GCG CGT CGC CGA CGG AGA AGG AAT AAC GAT GAC TGT TGC CAA CAG GAA GAG GGT GGC GGA GGG CAT CAC ATT ATC ATA ATG TTA TTG CTT CTC CTA CTG AAA AAG TTT TTC CCT CCC CCA CCG TCT TCC TCA TCG AGT AGC ACT ACC ACA ACG TGG TAT TAC GTT GTC GTA GTG TAA TGA TAG XXX
median expression values
#genes: 683
8
9
10
11
12
13
testset
bg

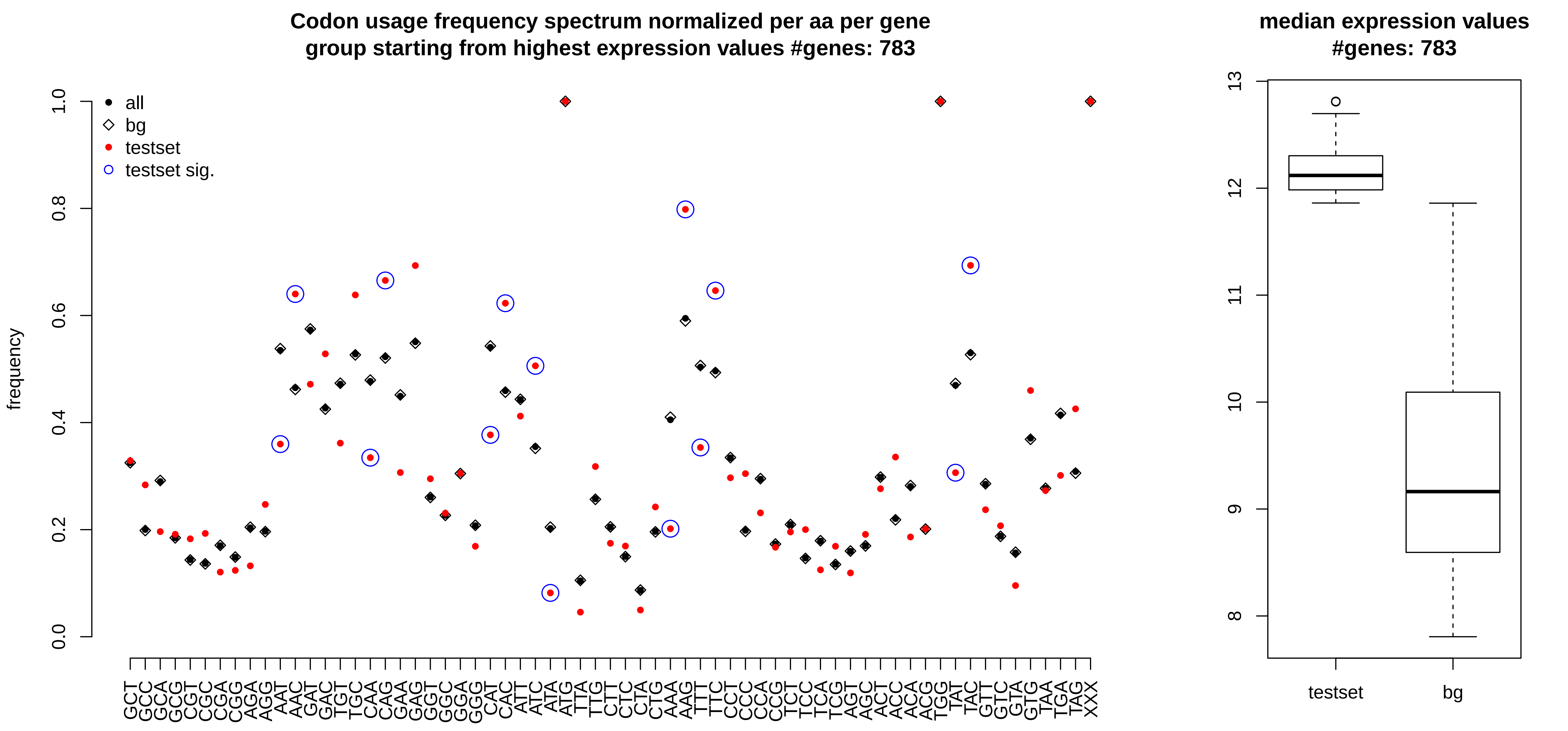

Codon usage frequency spectrum normalized per aa per gene
group starting from highest expression values #genes: 783
median expression values
#genes: 783
frequency
all
bg
testset
testset sig.
0.0
0.2
0.4
0.6
0.8
1.0
GCT GCC GCA GCG CGT CGC CGA CGG AGA AGG AAT AAC GAT GAC TGT TGC CAA CAG GAA GAG GGT GGC GGA GGG CAT CAC ATT ATC ATA ATG TTA TTG CTT CTC CTA CTG AAA AAG TTT TTC CCT CCC CCA CCG TCT TCC TCA TCG AGT AGC ACT ACC ACA ACG TGG TAT TAC GTT GTC GTA GTG TAA TGA TAG XXX
8
9
10
11
12
13
testset
bg

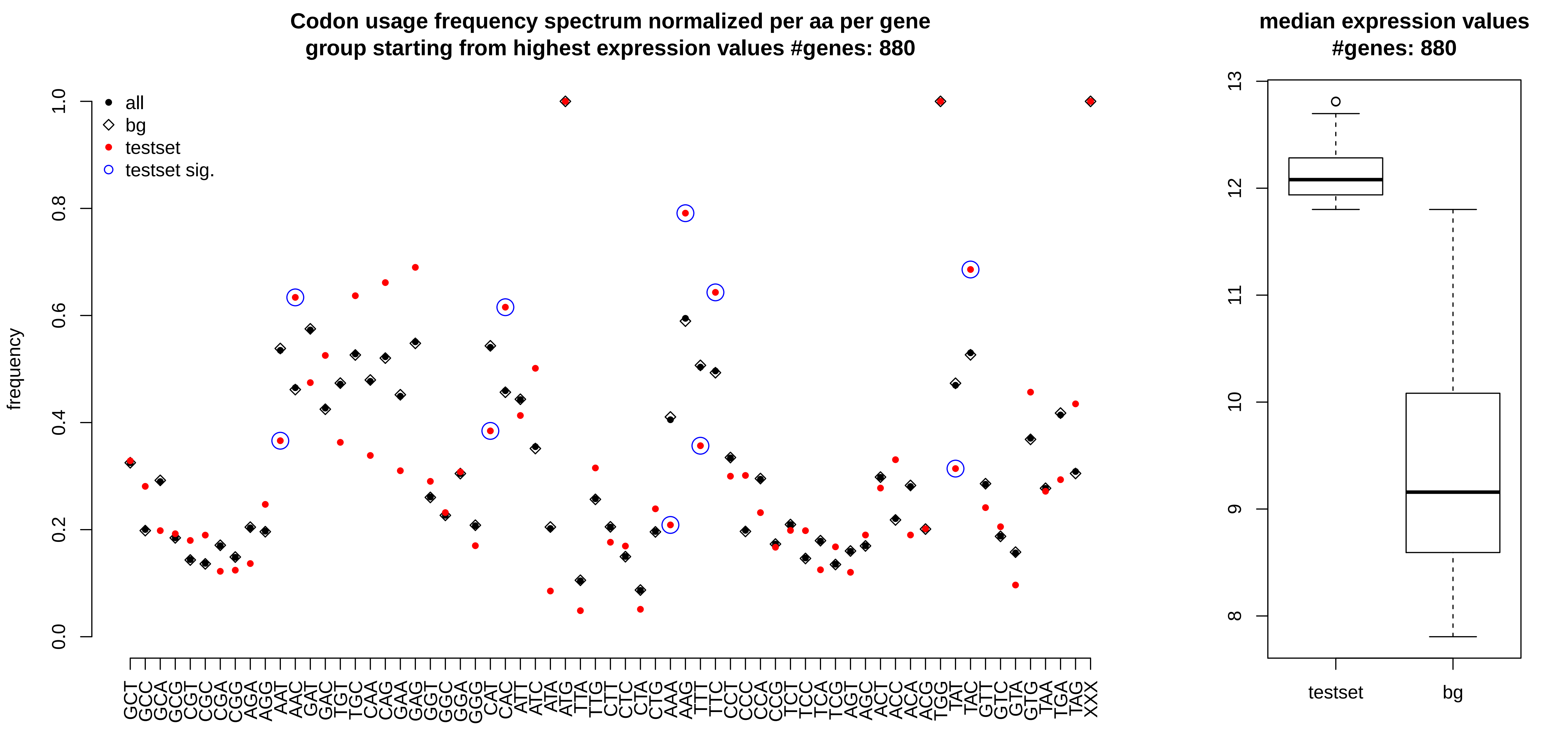

Codon usage frequency spectrum normalized per aa per gene
group starting from highest expression values #genes: 880
frequency
all
bg
testset
testset sig.
0.0
0.2
0.4
0.6
0.8
1.0
GCT GCC GCA GCG CGT CGC CGA CGG AGA AGG AAT AAC GAT GAC TGT TGC CAA CAG GAA GAG GGT GGC GGA GGG CAT CAC ATT ATC ATA ATG TTA TTG CTT CTC CTA CTG AAA AAG TTT TTC CCT CCC CCA CCG TCT TCC TCA TCG AGT AGC ACT ACC ACA ACG TGG TAT TAC GTT GTC GTA GTG TAA TGA TAG XXX
median expression values
#genes: 880
8
9
10
11
12
13
testset
bg

Codon usage frequency spectrum normalized per aa per gene group starting from highest expression values #genes: 980

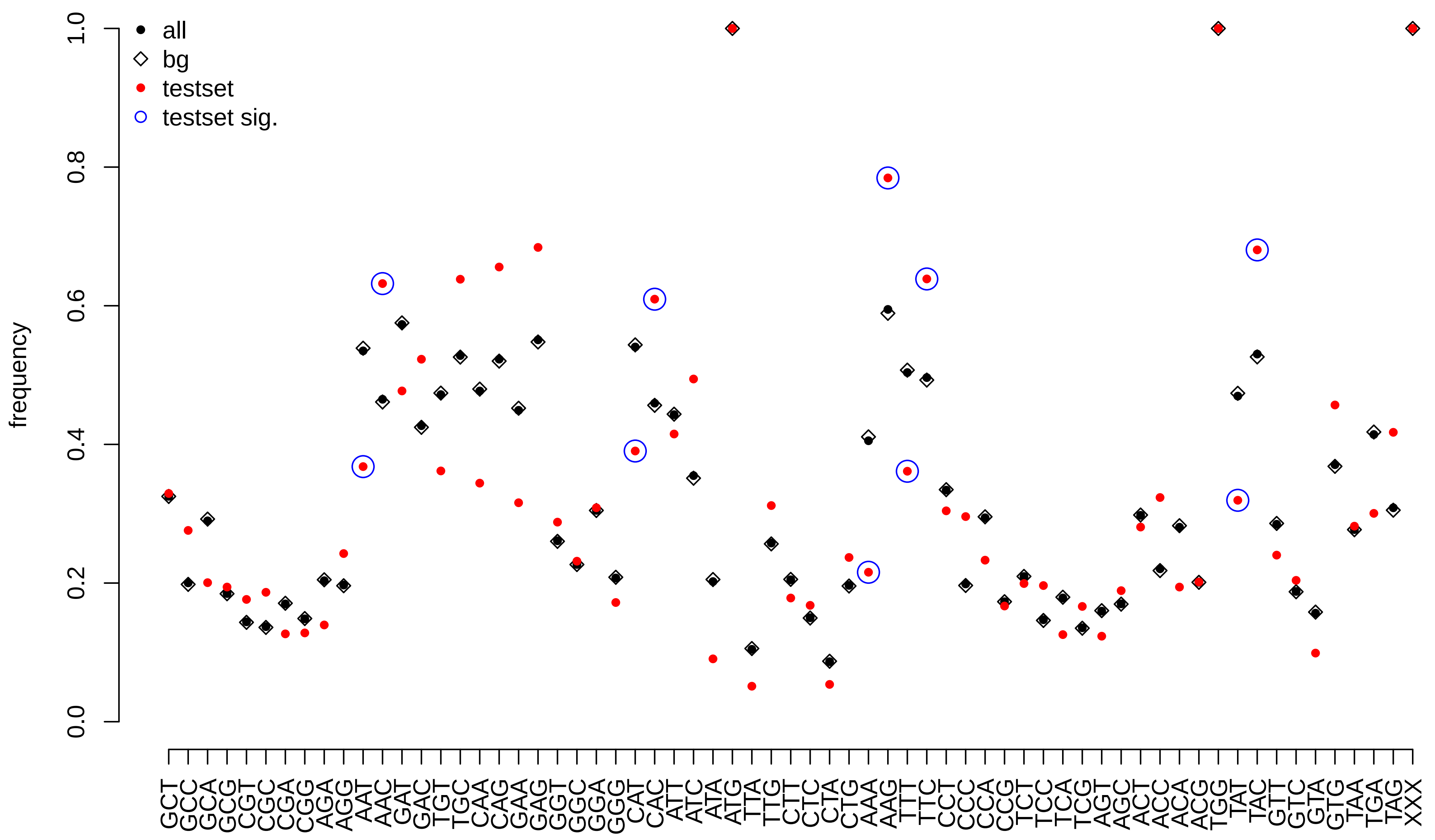

median expression values #genes: 980

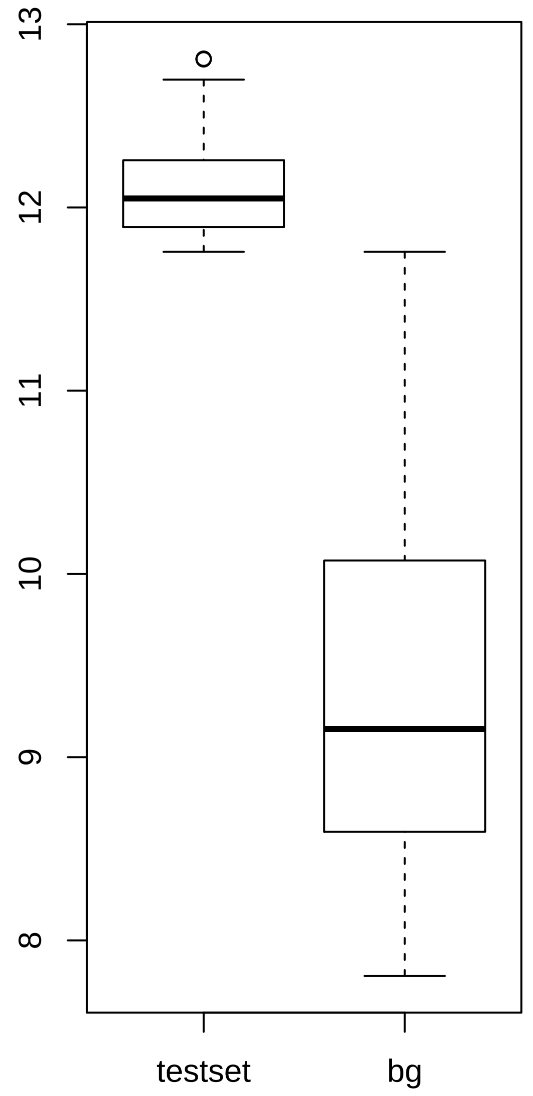

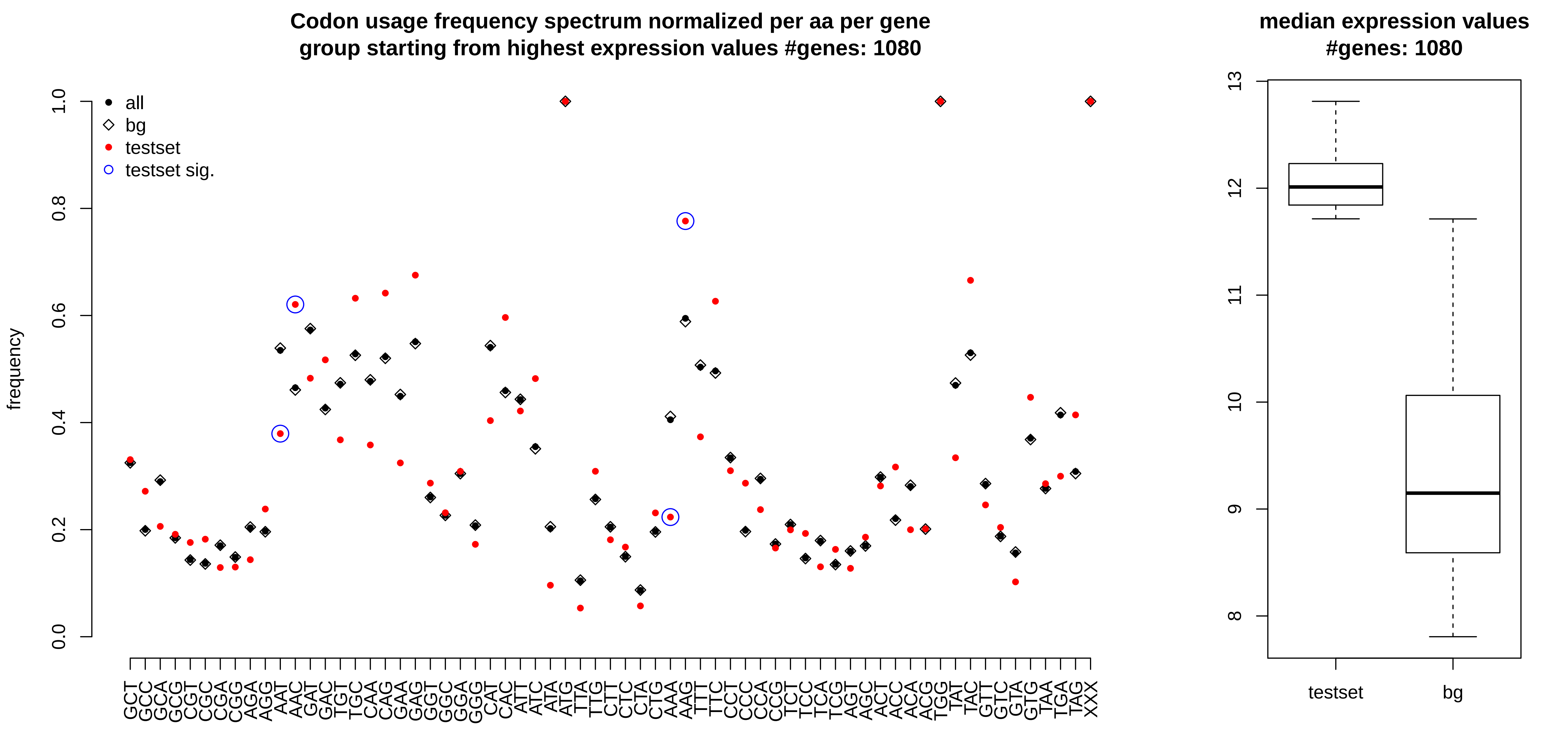
Codon usage frequency spectrum normalized per aa per gene group starting from highest expression values #genes: 1080
frequency
all
bg
testset
testset sig.
0.0
0.2
0.4
0.6
0.8
1.0
GCT GCC GCA GCG CGT CGC CGA CGG AGA AGG AAT AAC GAT GAC TGT TGC CAA CAG GAA GAG GGT GGC GGA GGG CAT CAC ATT ATC ATA ATG TTA TTG CTT CTC CTA CTG AAA AAG TTT TTC CCT CCC CCA CCG TCT TCC TCA TCG AGT AGC ACT ACC ACA ACG TGG TAT TAC GTT GTC GTA GTG TAA TGA TAG XXX
median expression values #genes: 1080
8
9
10
11
12
13
testset
bg

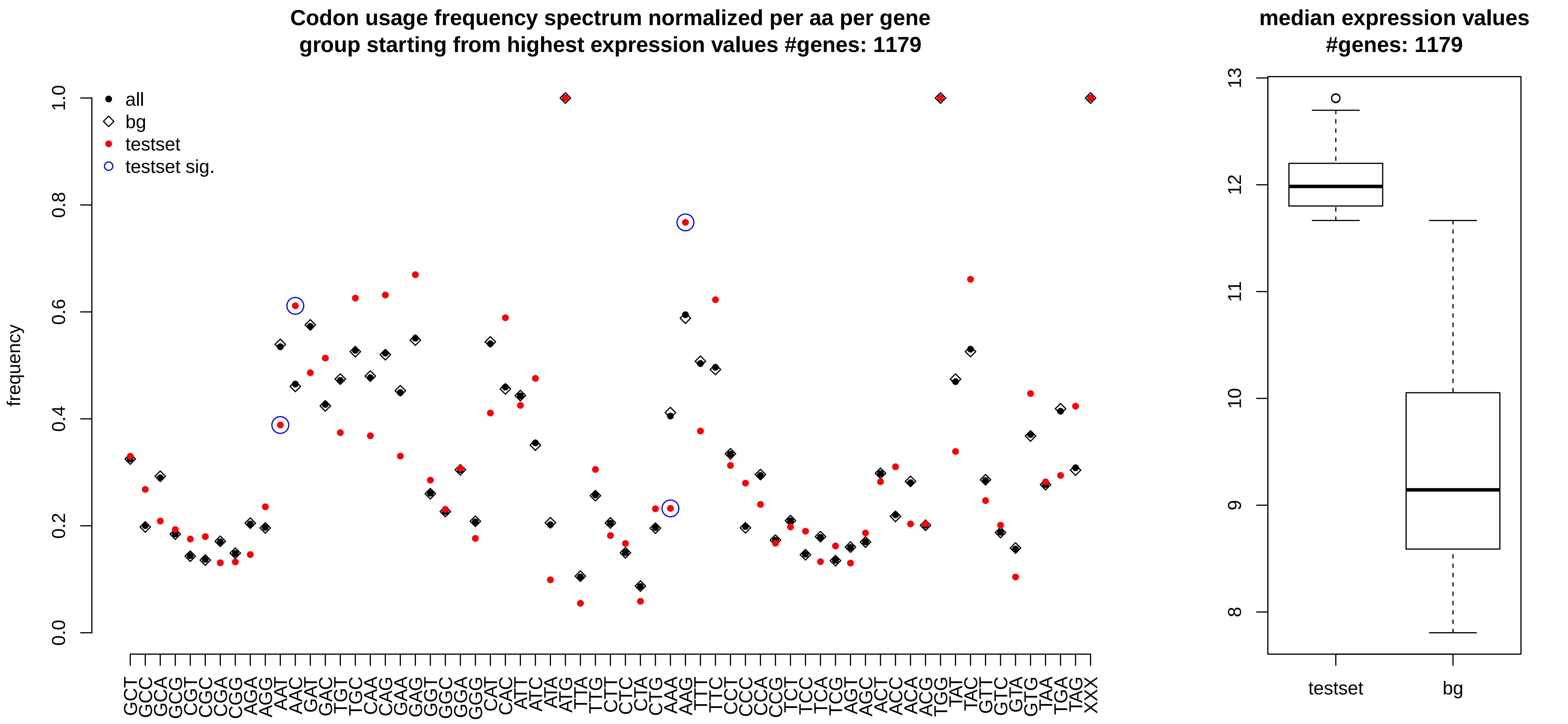
Codon usage frequency spectrum normalized per aa per gene
group starting from highest expression values #genes: 1179
frequency
0.0
0.2
0.4
0.6
0.8
1.0
all
bg
testset
testset sig.
GCT GCC GCA GCG CGT CGC CGA CGG AGA AGG AAT AAC GAT GAC TGT TGC CAA CAG GAA GAG GGT GGC GGA GGG CAT CAC ATT ATC ATA ATG TTA TTG CTT CTC CTA CTG AAA AAG TTT TTC CCT CCC CCA CCG TCT TCC TCA TCG AGT AGC ACT ACC ACA ACG TGG TAT TAC GTT GTC GTA GTG TAA TGA TAG XXX
median expression values
#genes: 1179
8
9
10
11
12
13
testset
bg

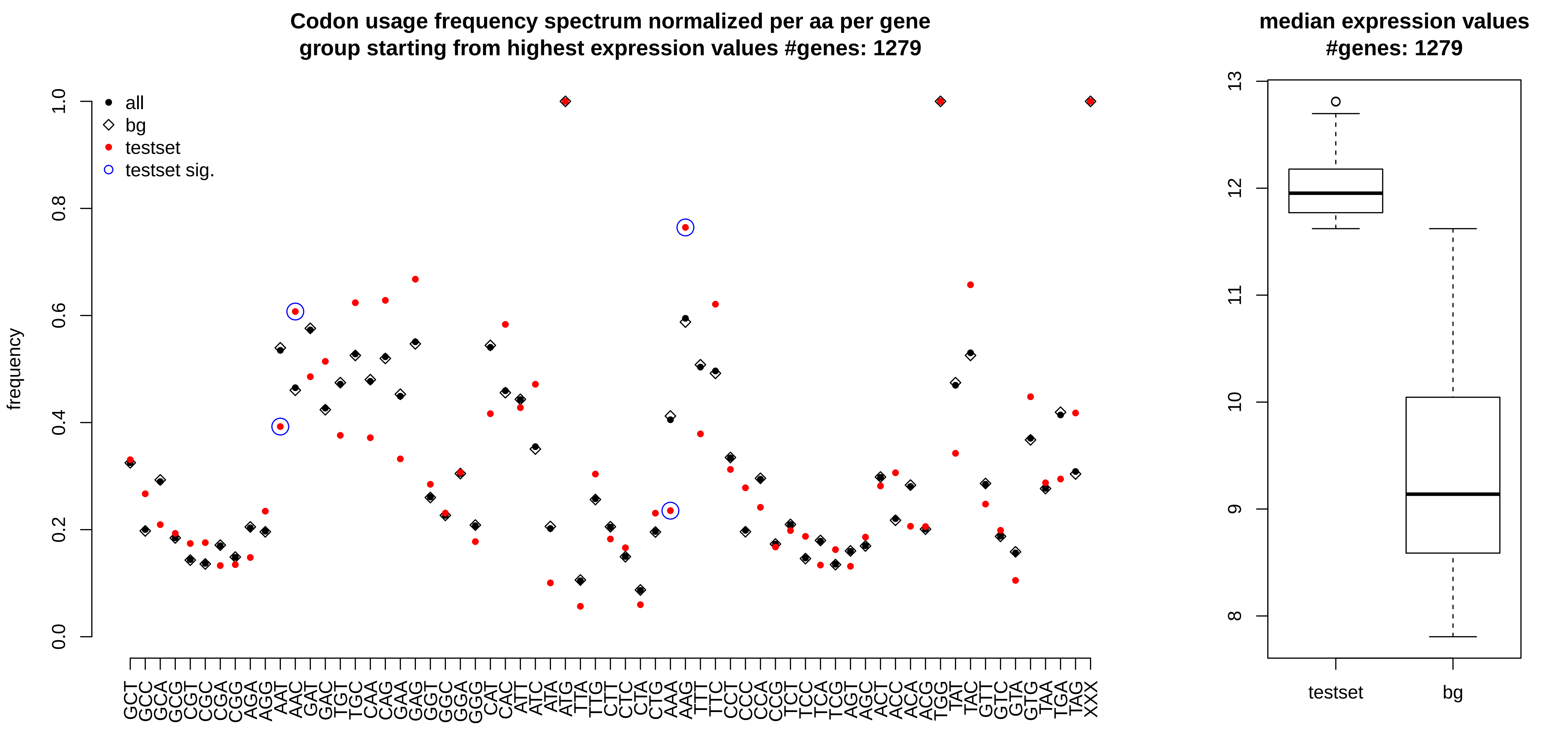
Codon usage frequency spectrum normalized per aa per gene
group starting from highest expression values #genes: 1279
frequency
all
bg
testset
testset sig.
0.0
0.2
0.4
0.6
0.8
1.0
GCT GCC GCA GCG CGT CGC CGA CGG AGA AGG AAT AAC GAT GAC TGT TGC CAA CAG GAA GAG GGT GGC GGA GGG CAT CAC ATT ATC ATA ATG TTA TTG CTT CTC CTA CTG AAA AAG TTT TTC CCT CCC CCA CCG TCT TCC TCA TCG AGT AGC ACT ACC ACA ACG TGG TAT TAC GTT GTC GTA GTG TAA TGA TAG XXX
median expression values
#genes: 1279
8
9
10
11
12
13
testset
bg

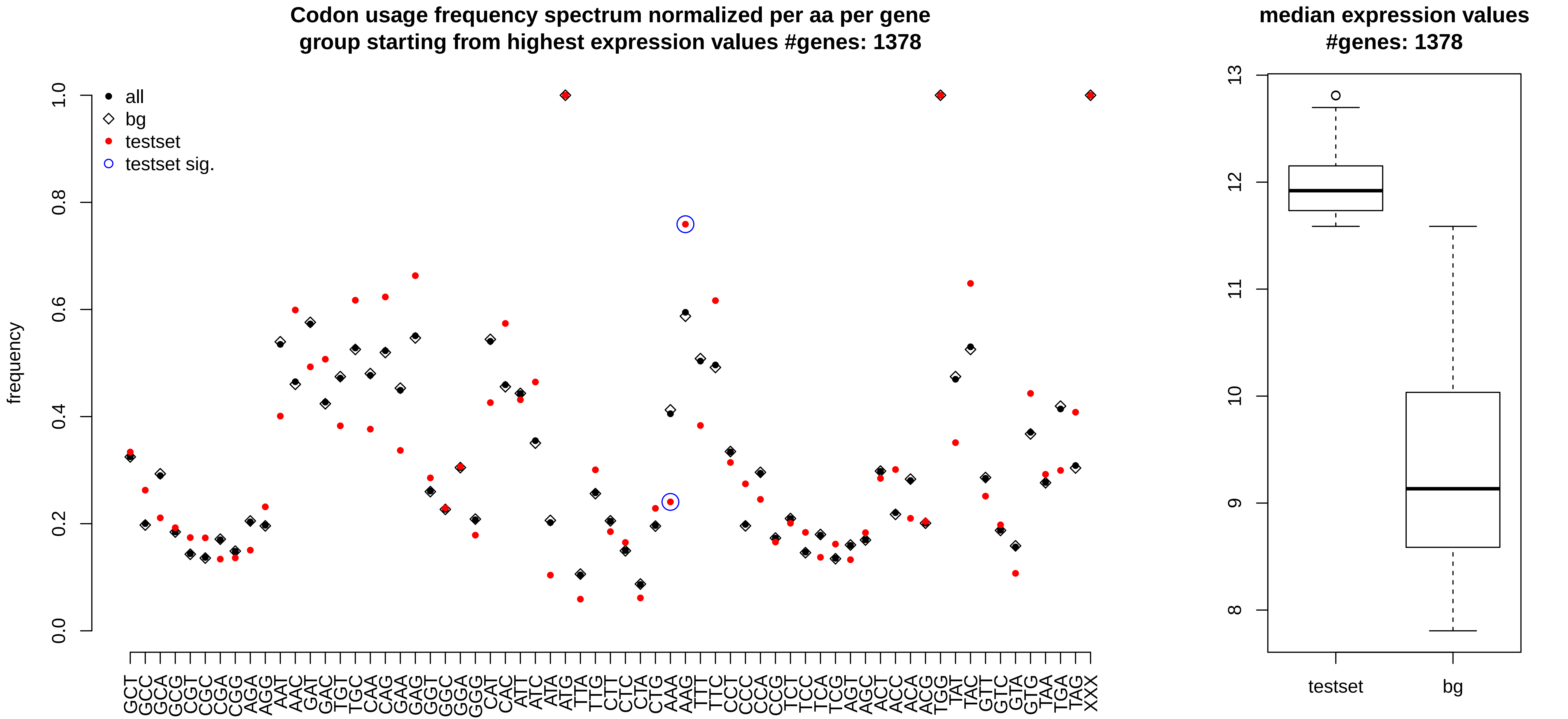

Codon usage frequency spectrum normalized per aa per gene
group starting from highest expression values #genes: 1378
frequency
all
bg
testset
testset sig.
0.0
0.2
0.4
0.6
0.8
1.0
GCT GCC GCA GCG CGT CGC CGA CGG AGA AGG AAT AAC GAT GAC TGT TGC CAA CAG GAA GAG GGT GGC GGA GGG CAT CAC ATT ATC ATA ATG TTA TTG CTT CTC CTA CTG AAA AAG TTT TTC CCT CCC CCA CCG TCT TCC TCA TCG AGT AGC ACT ACC ACA ACG TGG TAT TAC GTT GTC GTA GTG TAA TGA TAG XXX
median expression values
#genes: 1378
8
9
10
11
12
13
testset
bg

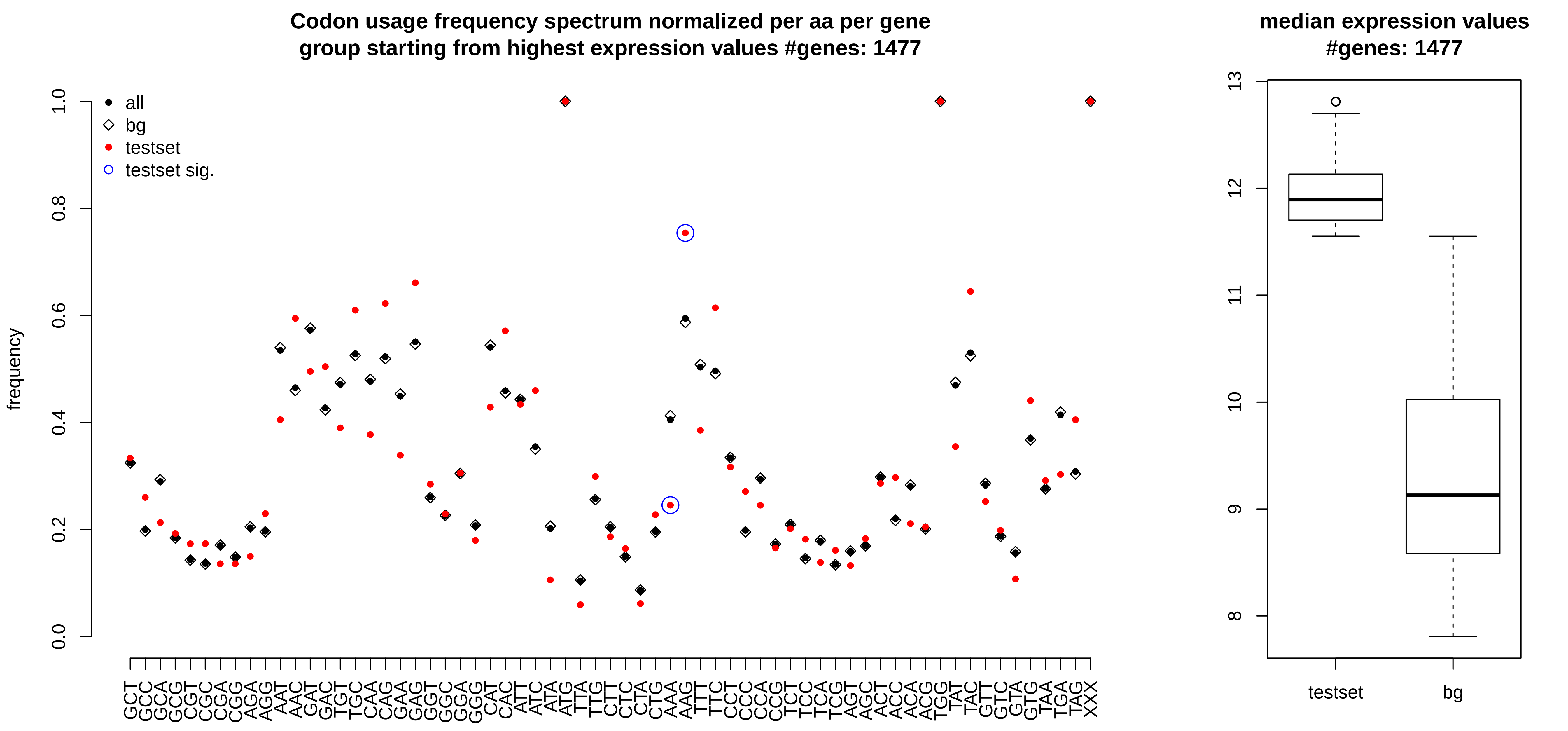

Codon usage frequency spectrum normalized per aa per gene
group starting from highest expression values #genes: 1477
frequency
all
bg
testset
testset sig.
0.0
0.2
0.4
0.6
0.8
1.0
GCT GCC GCA GCG CGT CGC CGA CGG AGA AGG AAT AAC GAT GAC TGT TGC CAA CAG GAA GAG GGT GGC GGA GGG CAT CAC ATT ATC ATA ATG TTA TTG CTT CTC CTA CTG AAA AAG TTT TTC CCT CCC CCA CCG TCT TCC TCA TCG AGT AGC ACT ACC ACA ACG TGG TAT TAC GTT GTC GTA GTG TAA TGA TAG XXX
median expression values
#genes: 1477
8
9
10
11
12
13
testset
bg

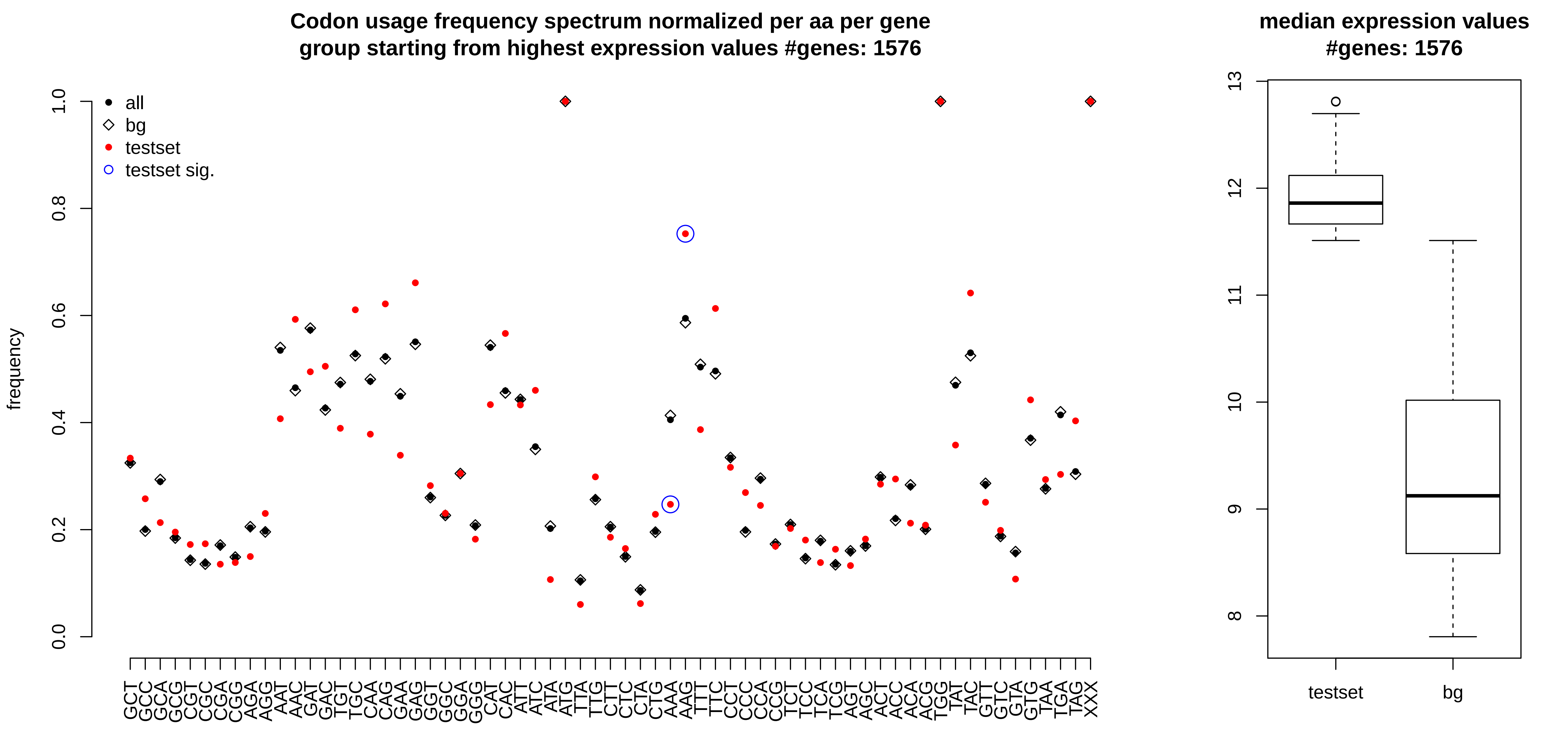

Codon usage frequency spectrum normalized per aa per gene
group starting from highest expression values #genes: 1576
frequency
0.0
0.2
0.4
0.6
0.8
1.0
all
bg
testset
testset sig.
GCT GCC GCA GCG CGT CGC CGA CGG AGA AGG AAT AAC GAT GAC TGT TGC CAA CAG GAA GAG GGT GGC GGA GGG CAT CAC ATT ATC ATA ATG TTA TTG CTT CTC CTA CTG AAA AAG TTT TTC CCT CCC CCA CCG TCT TCC TCA TCG AGT AGC ACT ACC ACA ACG TGG TAT TAC GTT GTC GTA GTG TAA TGA TAG XXX
median expression values
#genes: 1576
8
9
10
11
12
13
testset
bg

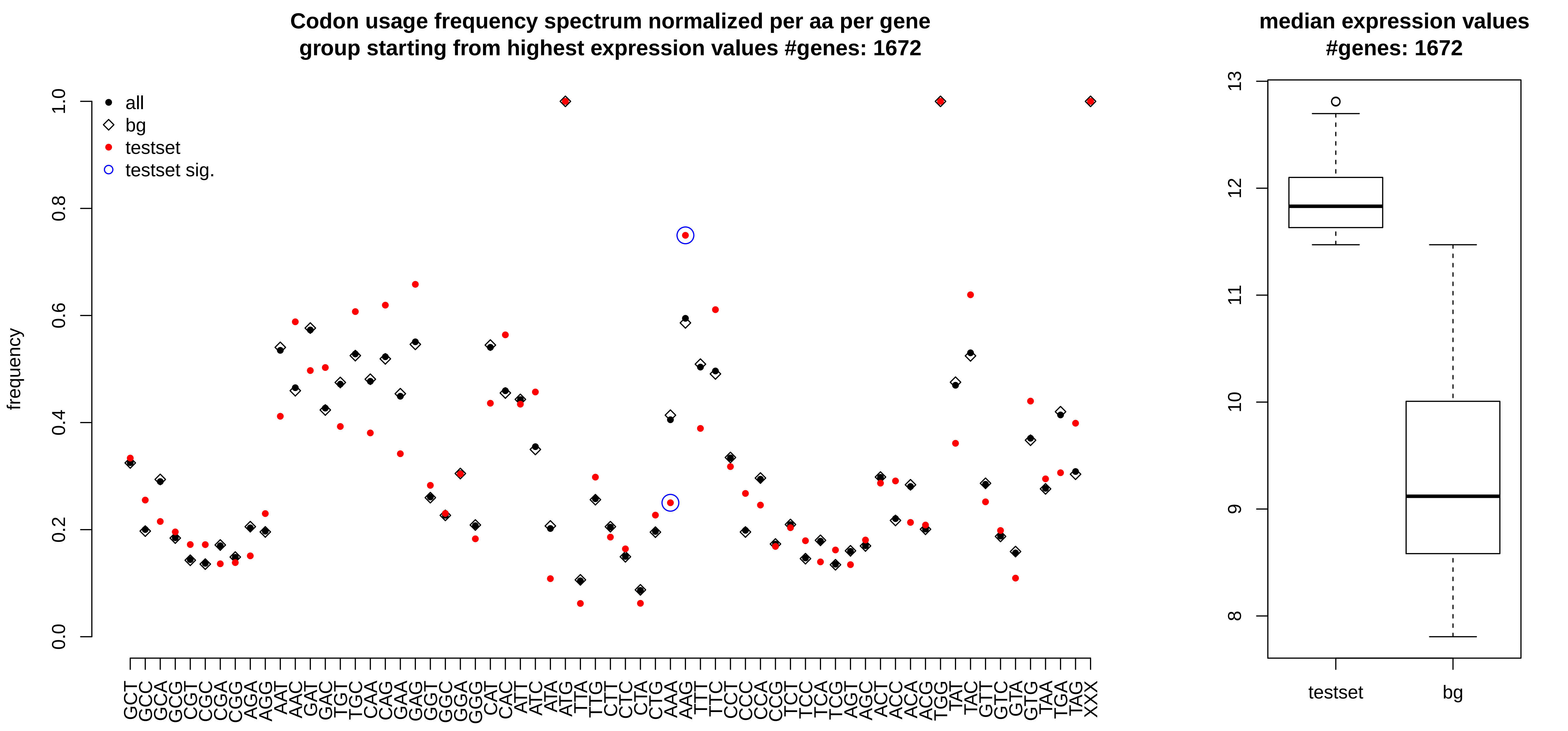

Codon usage frequency spectrum normalized per aa per gene
group starting from highest expression values #genes: 1672
frequency
0.0
0.2
0.4
0.6
0.8
1.0
all
bg
testset
testset sig.
GCT GCC GCA GCG CGT CGC CGA CGG AGA AGG AAT AAC GAT GAC TGT TGC CAA CAG GAA GAG GGT GGC GGA GGG CAT CAC ATT ATC ATA ATG TTA TTG CTT CTC CTA CTG AAA AAG TTT TTC CCT CCC CCA CCG TCT TCC TCA TCG AGT AGC ACT ACC ACA ACG TGG TAT TAC GTT GTC GTA GTG TAA TGA TAG XXX
median expression values
#genes: 1672
8
9
10
11
12
13
testset
bg

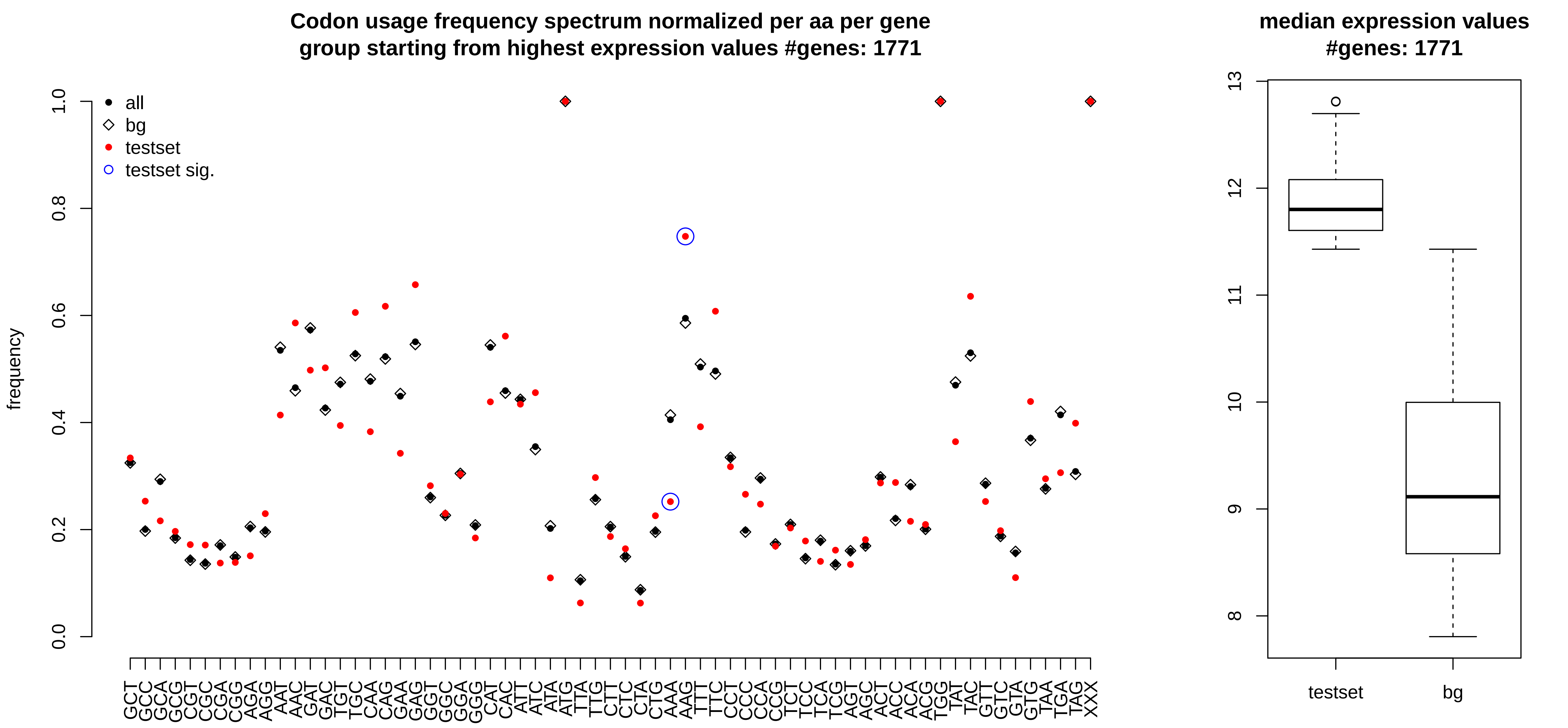

Codon usage frequency spectrum normalized per aa per gene group starting from highest expression values #genes: 1771
frequency
all
bg
testset
testset sig.
0.0
0.2
0.4
0.6
0.8
1.0
GCT GCC GCA GCG CGT CGC CGA CGG AGA AGG AAT AAC GAT GAC TGT TGC CAA CAG GAA GAG GGT GGC GGA GGG CAT CAC ATT ATC ATA ATG TTA TTG CTT CTC CTA CTG AAA AAG TTT TTC CCT CCC CCA CCG TCT TCC TCA TCG AGT AGC ACT ACC ACA ACG TGG TAT TAC GTT GTC GTA GTG TAA TGA TAG XXX
median expression values #genes: 1771
8
9
10
11
12
13
testset
bg

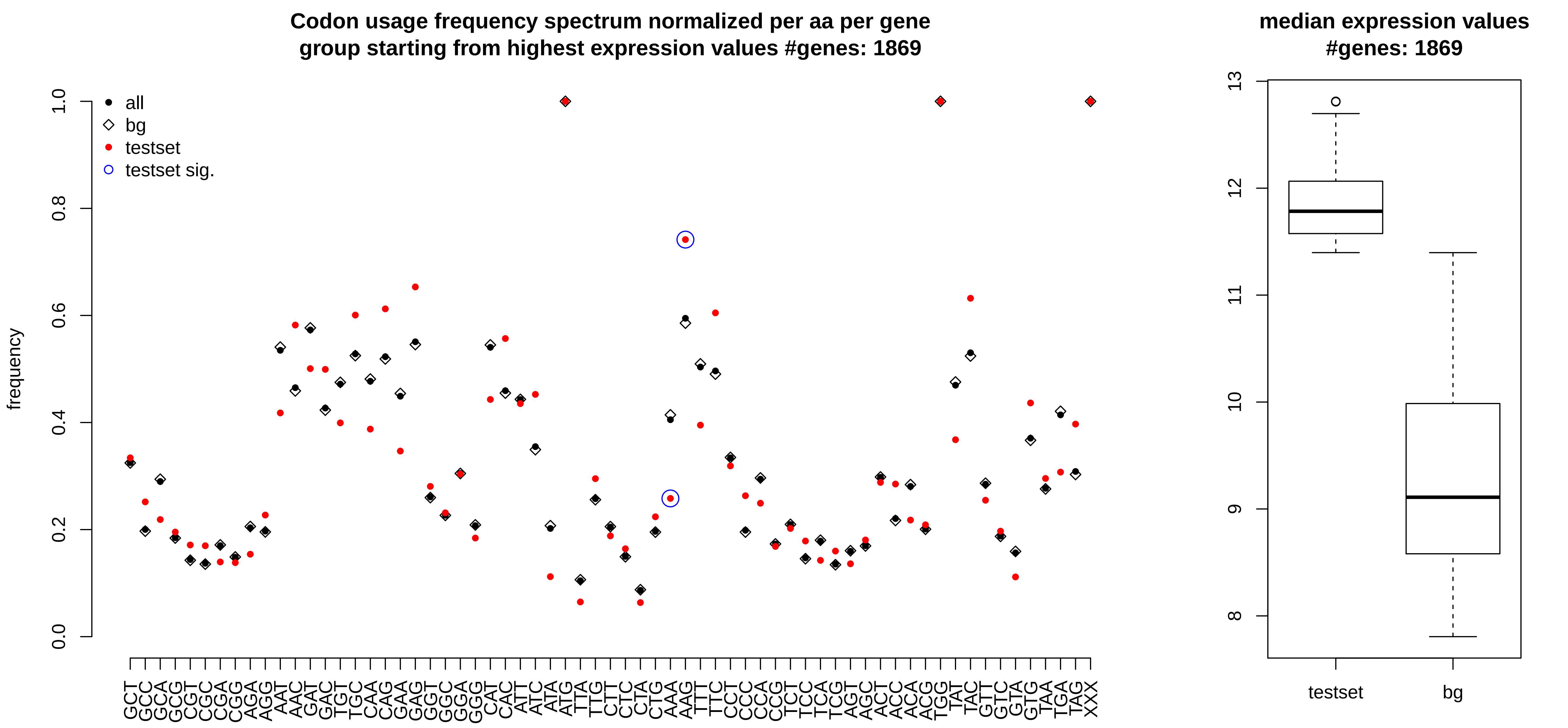

Codon usage frequency spectrum normalized per aa per gene
group starting from highest expression values #genes: 1869
frequency
0.0
0.2
0.4
0.6
0.8
1.0
all
bg
testset
testset sig.
GCT GCC GCA GCG CGT CGC CGA CGG AGA AGG AAT AAC GAT GAC TGT TGC CAA CAG GAA GAG GGT GGC GGA GGG CAT CAC ATT ATC ATA ATG TTA TTG CTT CTC CTA CTG AAA AAG TTT TTC CCT CCC CCA CCG TCT TCC TCA TCG AGT AGC ACT ACC ACA ACG TGG TAT TAC GTT GTC GTA GTG TAA TGA TAG XXX
median expression values
#genes: 1869
8
9
10
11
12
13
testset
bg

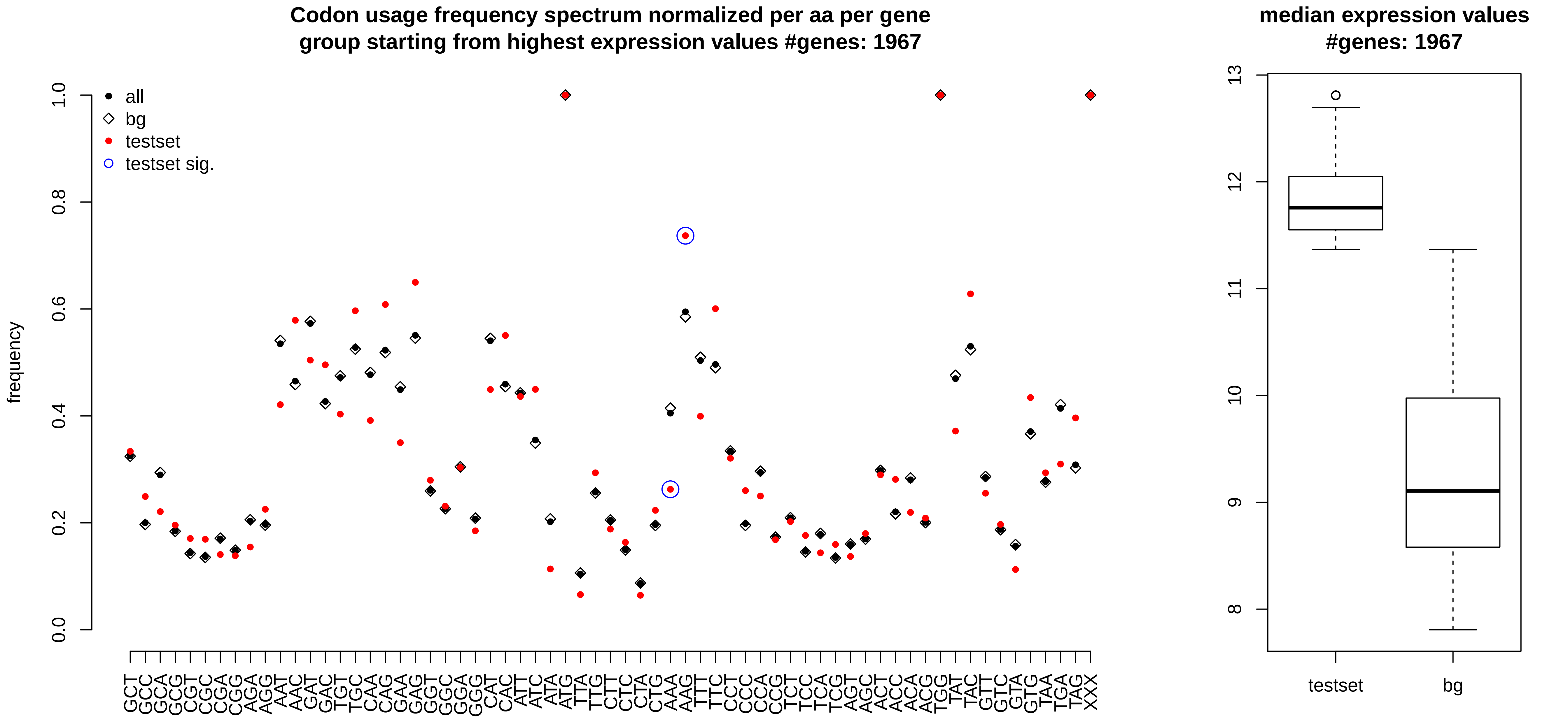

Codon usage frequency spectrum normalized per aa per gene
group starting from highest expression values #genes: 1967
frequency
all
bg
testset
testset sig.
0.0
0.2
0.4
0.6
0.8
1.0
GCT GCC GCA GCG CGT CGC CGA CGG AGA AGG AAT AAC GAT GAC TGT TGC CAA CAG GAA GAG GGT GGC GGA GGG CAT CAC ATT ATC ATA ATG TTA TTG CTT CTC CTA CTG AAA AAG TTT TTC CCT CCC CCA CCG TCT TCC TCA TCG AGT AGC ACT ACC ACA ACG TGG TAT TAC GTT GTC GTA GTG TAA TGA TAG XXX
median expression values
#genes: 1967
8
9
10
11
12
13
testset
bg

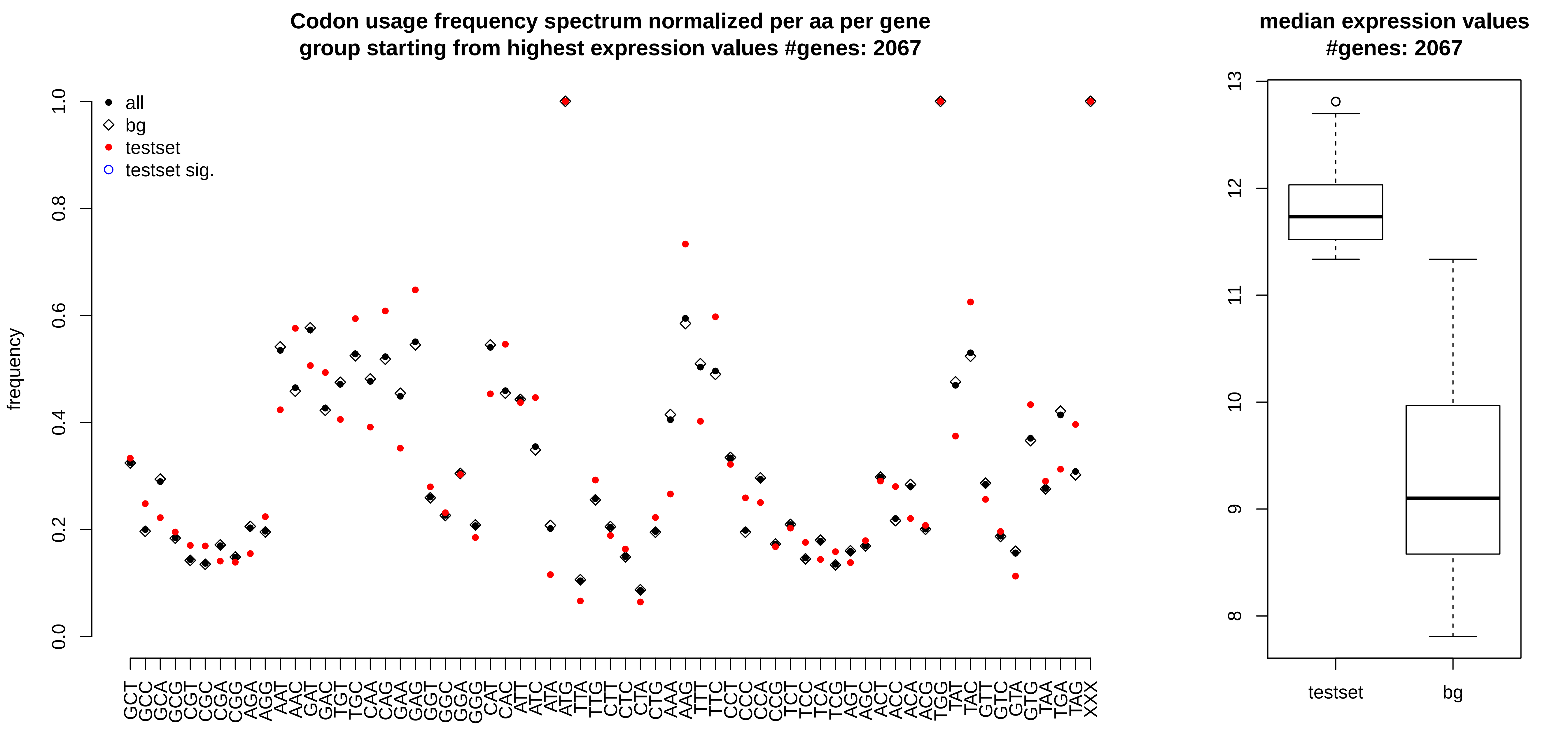

Codon usage frequency spectrum normalized per aa per gene
group starting from highest expression values #genes: 2067
frequency
all
bg
testset
testset sig.
0.0
0.2
0.4
0.6
0.8
1.0
GCT GCC GCA GCG CGT CGC CGA CGG AGA AGG AAT AAC GAT GAC TGT TGC CAA CAG GAA GAG GGT GGC GGA GGG CAT CAC ATT ATC ATA ATG TTA TTG CTT CTC CTA CTG AAA AAG TTT TTC CCT CCC CCA CCG TCT TCC TCA TCG AGT AGC ACT ACC ACA ACG TGG TAT TAC GTT GTC GTA GTG TAA TGA TAG XXX
median expression values
#genes: 2067
8
9
10
11
12
13
testset
bg

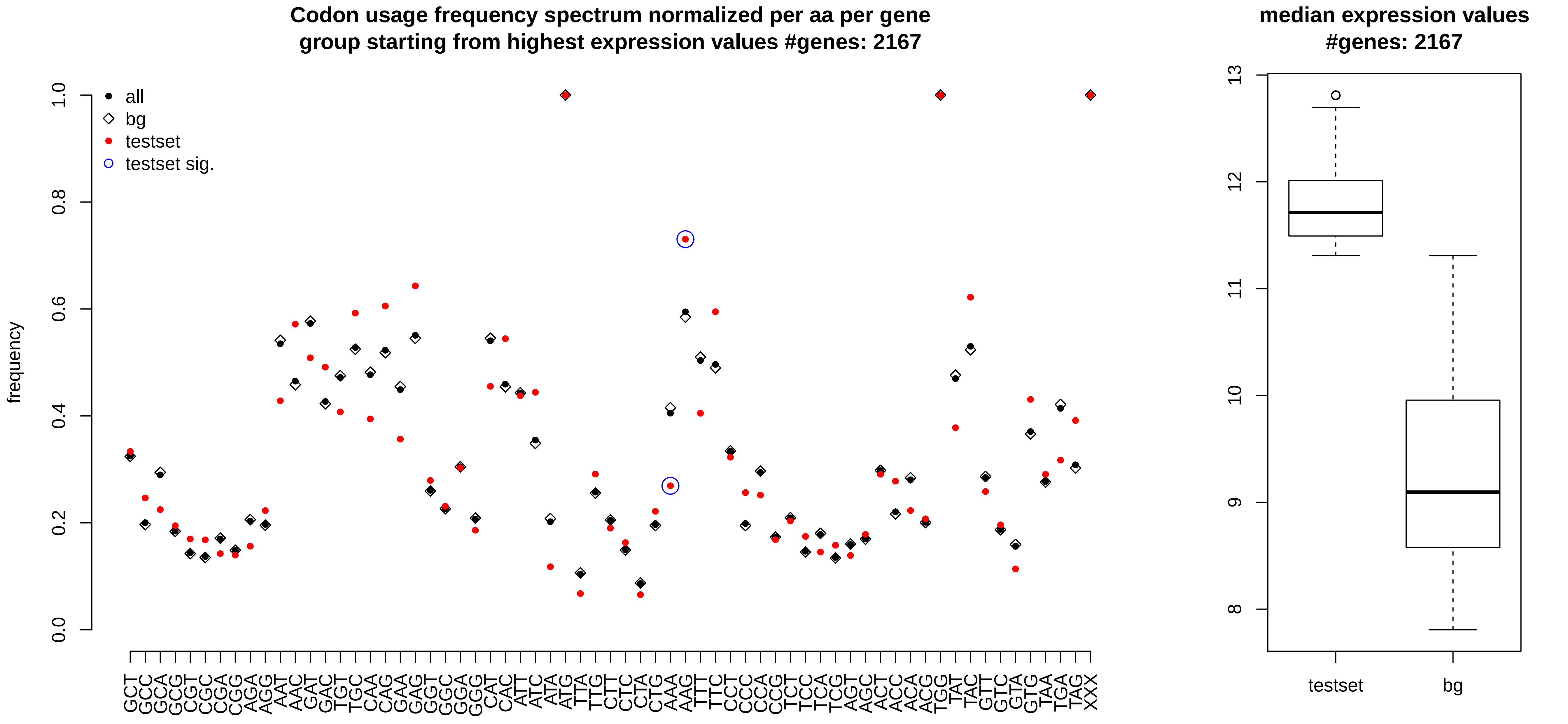

Codon usage frequency spectrum normalized per aa per gene
group starting from highest expression values #genes: 2167
frequency
all
bg
testset
testset sig.
0.0
0.2
0.4
0.6
0.8
1.0
GCT GCC GCA GCG CGT CGC CGA CGG AGA AGG AAT AAC GAT GAC TGT TGC CAA CAG GAA GAG GGT GGC GGA GGG CAT CAC ATT ATC ATA ATG TTA TTG CTT CTC CTA CTG AAA AAG TTT TTC CCC CCA CCG TCT TCC TCA TCG AGT AGC ACT ACC ACA ACG TGG TAT TAC GTT GTC GTA GTG TAA TGA TAG XXX
median expression values
#genes: 2167
8
9
10
11
12
13
testset
bg

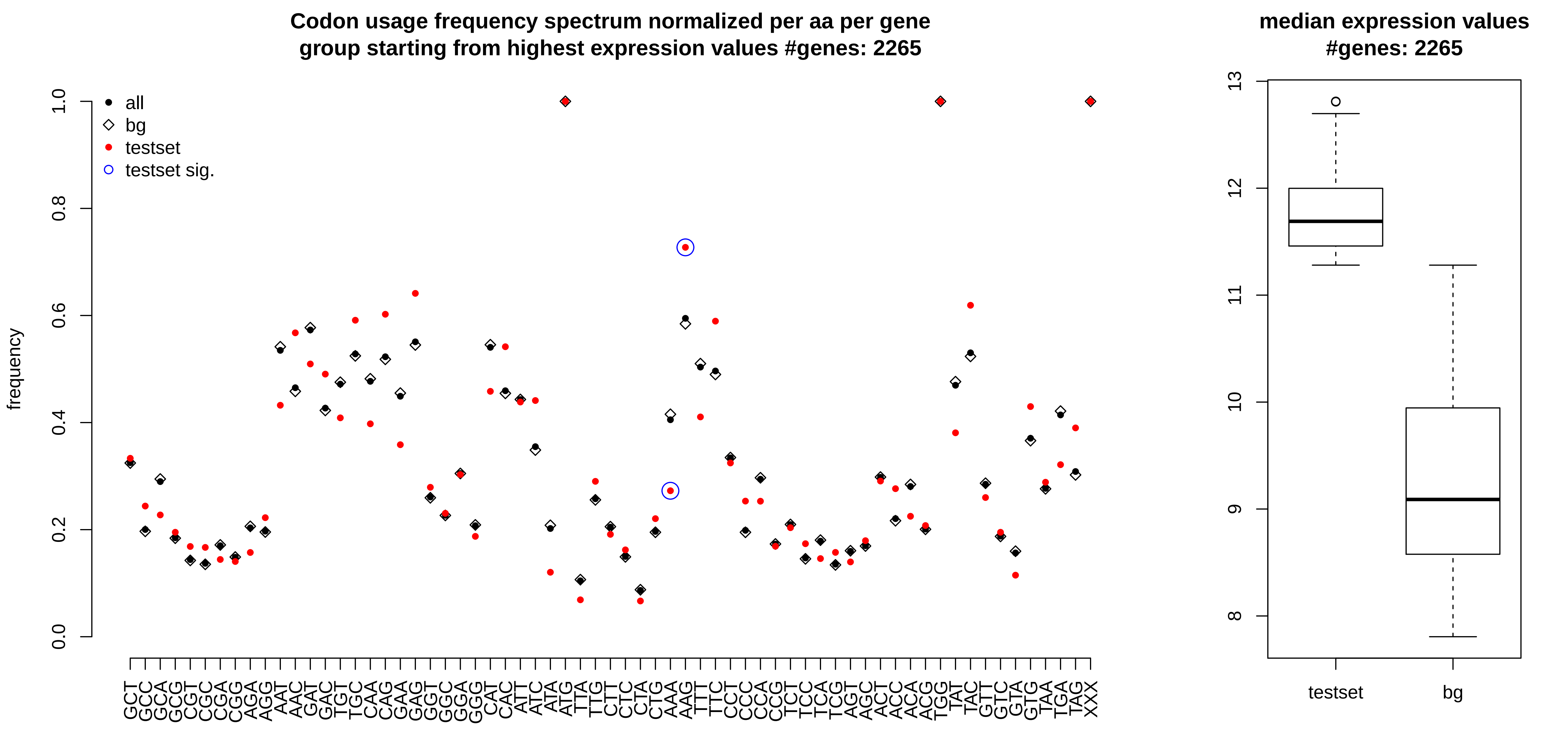

Codon usage frequency spectrum normalized per aa per gene
group starting from highest expression values #genes: 2265
frequency
all
bg
testset
testset sig.
0.0
0.2
0.4
0.6
0.8
1.0
GCT GCC GCA GCG CGT CGC CGA CGG AGA AGG AAT AAC GAT GAC TGT TGC CAA CAG GAA GAG GGT GGC GGA GGG CAT CAC ATT ATC ATA ATG TTA TTG CTT CTC CTA CTG AAA AAG TTT TTC CCT CCC CCA CCG TCT TCC TCA TCG AGT AGC ACT ACC ACA ACG TGG TAT TAC GTT GTC GTA GTG TAA TGA TAG XXX
median expression values
#genes: 2265
8
9
10
11
12
13
testset
bg

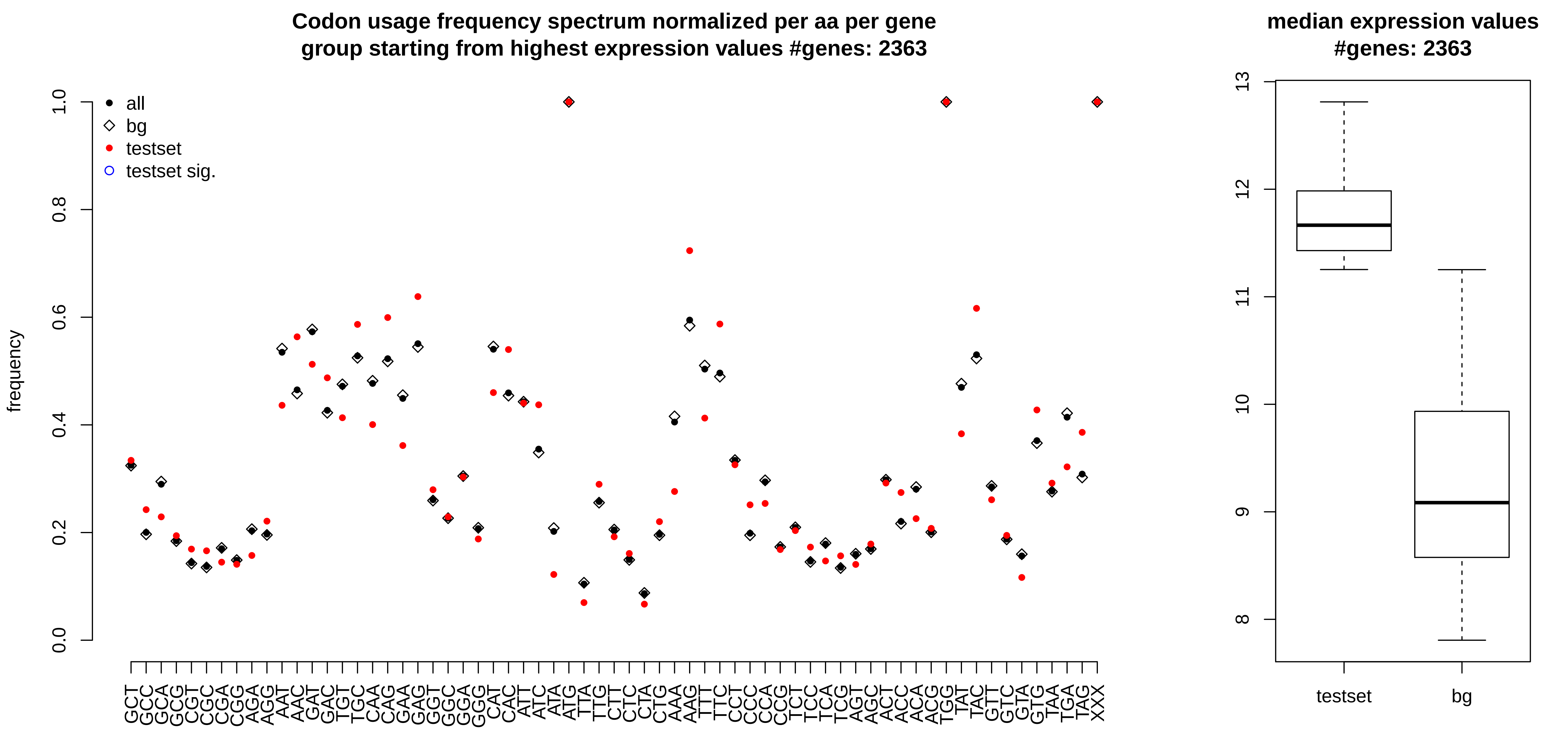

Codon usage frequency spectrum normalized per aa per gene
group starting from highest expression values #genes: 2363
frequency
0.0
0.2
0.4
0.6
0.8
1.0
all
bg
testset
testset sig.
GCT GCC GCA GCG CGT CGC CGA CGG AGA AGG AAT AAC GAT GAC TGT TGC CAA CAG GAA GAG GGT GGC GGA GGG CAT CAC ATT ATC ATA ATG TTA TTG CTT CTC CTA CTG AAA AAG TTT TTC CCT CCC CCA CCG TCT TCC TCA TCG AGT AGC ACT ACC ACA ACG TGG TAT TAC GTT GTC GTA GTG TAA TGA TAG XXX
median expression values
#genes: 2363
8
9
10
11
12
13
testset
bg

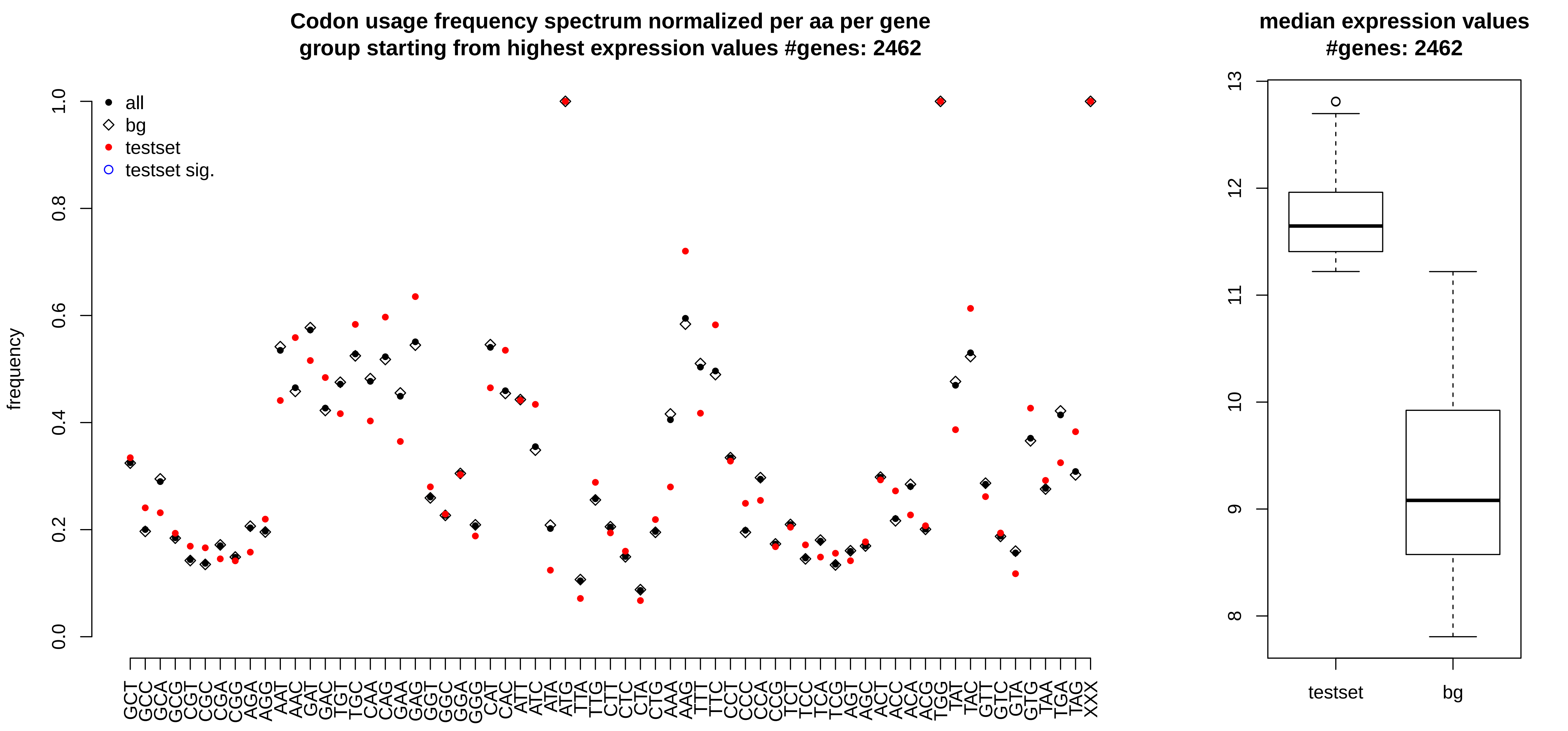

Codon usage frequency spectrum normalized per aa per gene
group starting from highest expression values #genes: 2462
frequency
0.0
0.2
0.4
0.6
0.8
1.0
all
bg
testset
testset sig.
GCT GCC GCA GCG CGT CGC CGA CGG AGA AGG AAT AAC GAT GAC TGT TGC CAA CAG GAA GAG GGT GGC GGA GGG CAT CAC ATT ATC ATA ATG TTA TTG CTT CTC CTA CTG AAA AAG TTT TTC CCT CCC CCA CCG TCT TCC TCA TCG AGT AGC ACT ACC ACA ACG TGG TAT TAC GTT GTC GTA GTG TAA TGA TAG XXX
median expression values
#genes: 2462
8
9
10
11
12
13
testset
bg

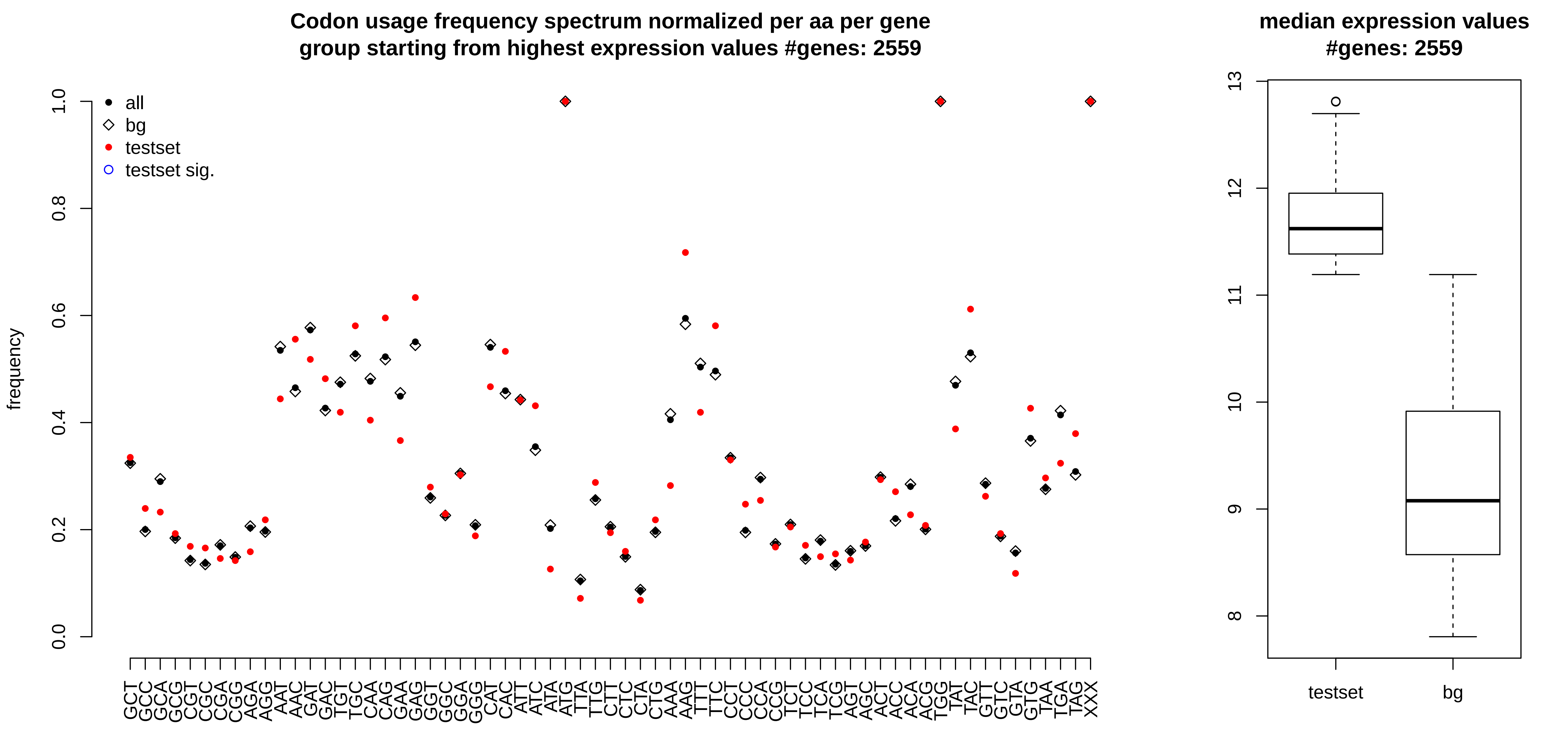

Codon usage frequency spectrum normalized per aa per gene
group starting from highest expression values #genes: 2559
frequency
all
bg
testset
testset sig.
0.0
0.2
0.4
0.6
0.8
1.0
GCT GCC GCA GCG CGT CGC CGA CGG AGA AGG AAT AAC GAT GAC TGT TGC CAA CAG GAA GAG GGT GGC GGA GGG CAT CAC ATT ATC ATA ATG TTA TTG CTT CTC CTA CTG AAA AAG TTT TTC CCT CCC CCA CCG TCT TCC TCA TCG AGT AGC ACT ACC ACA ACG TGG TAT TAC GTT GTC GTA GTG TAA TGA TAG XXX
median expression values
#genes: 2559
8
9
10
11
12
13
testset
bg

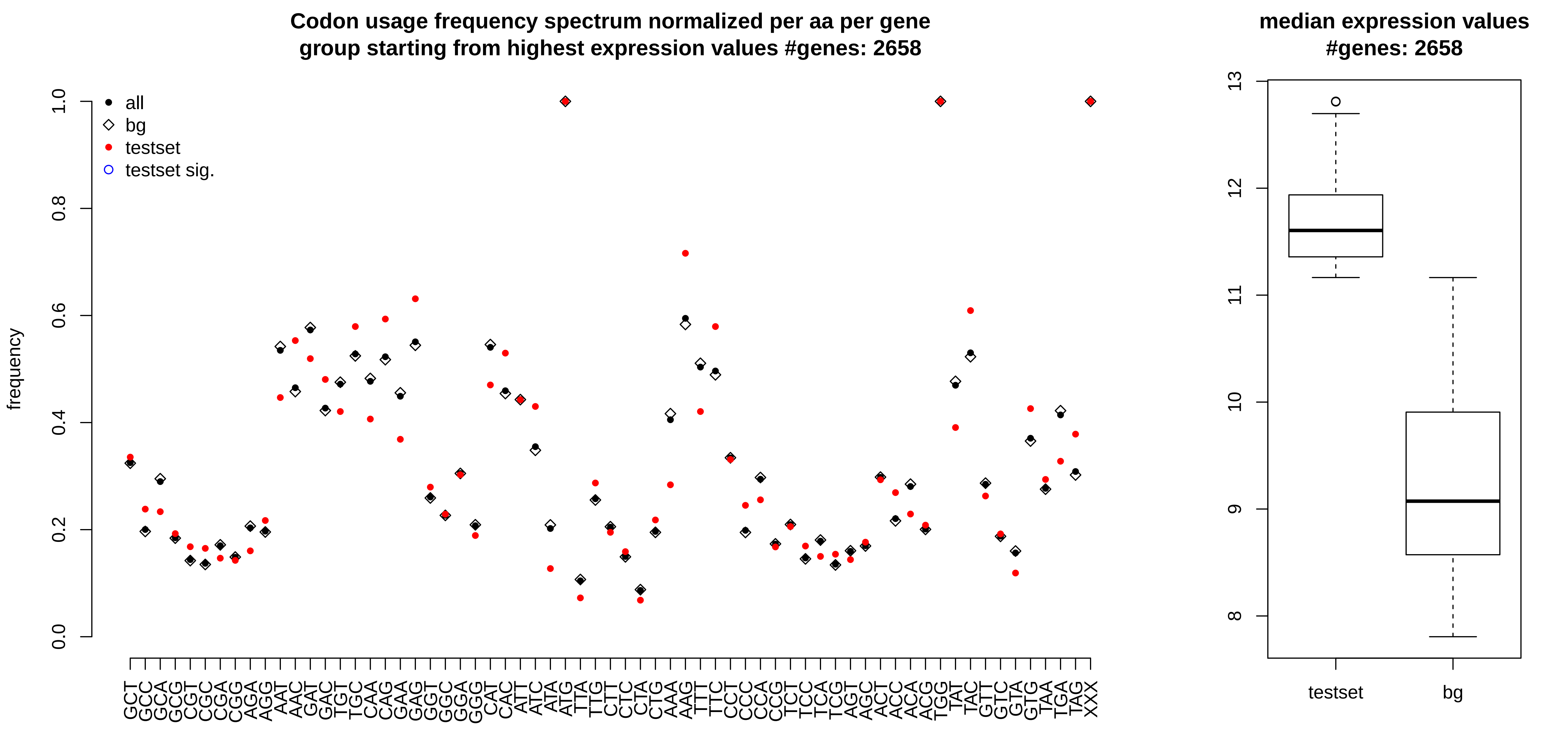
Codon usage frequency spectrum normalized per aa per gene
group starting from highest expression values #genes: 2658
frequency
all
bg
testset
testset sig.
median expression values
#genes: 2658
testset
bg

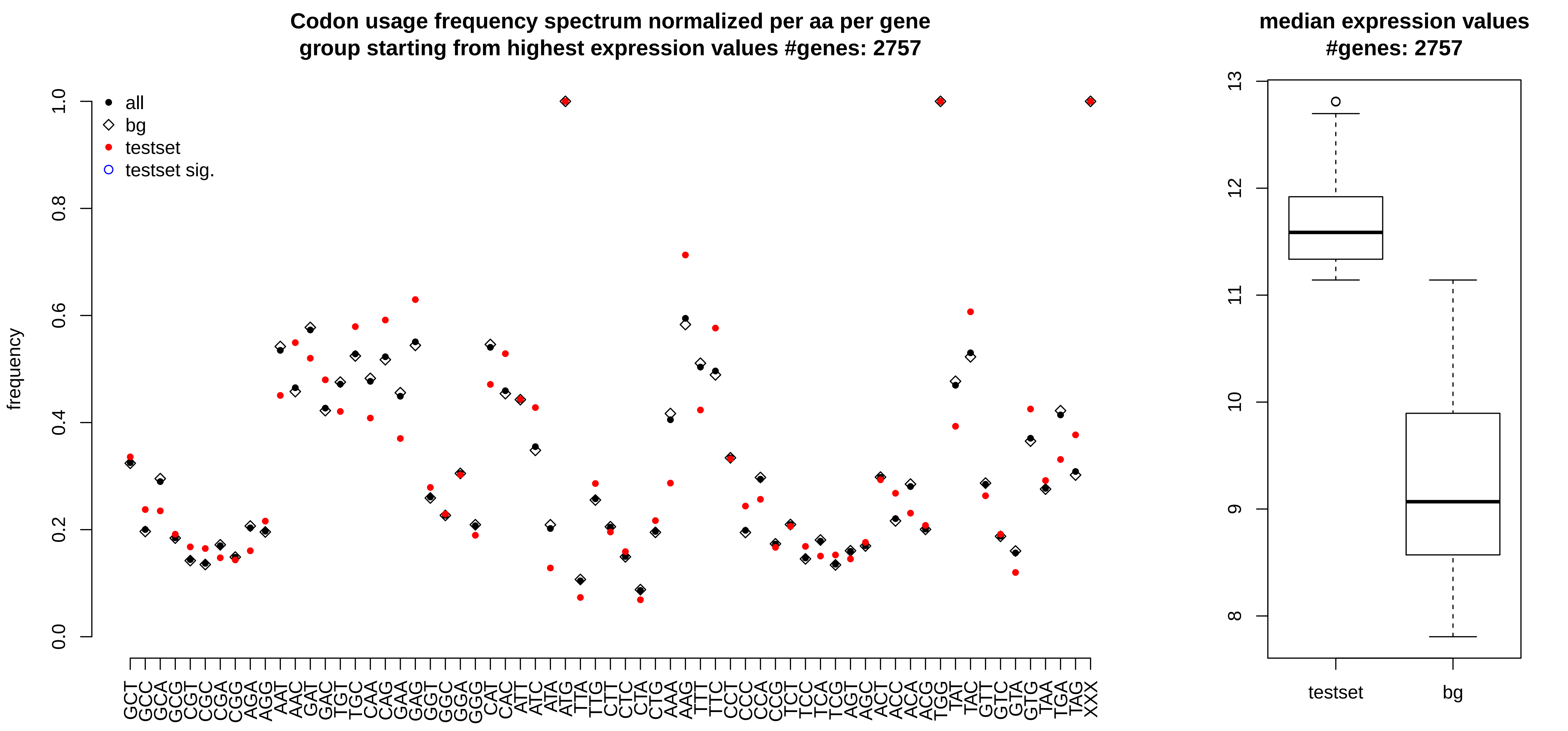

Codon usage frequency spectrum normalized per aa per gene group starting from highest expression values #genes: 2757
frequency
all
bg
testset
testset sig.
0.0
0.2
0.4
0.6
0.8
1.0
GCT GCC GCA GCG CGT CGC CGA CGG AGA AGG AAT AAC GAT GAC TGT TGC CAA CAG GAA GAG GGT GGC GGA GGG CAT CAC ATT ATC ATA ATG TTA TTG CTT CTC CTA CTG AAA AAG TTT TTC CCT CCC CCA CCG TCT TCC TCA TCG AGT AGC ACT ACC ACA ACG TGG TAT TAC GTT GTC GTA GTG TAA TGA TAG XXX
median expression values #genes: 2757
8
9
10
11
12
13
testset
bg

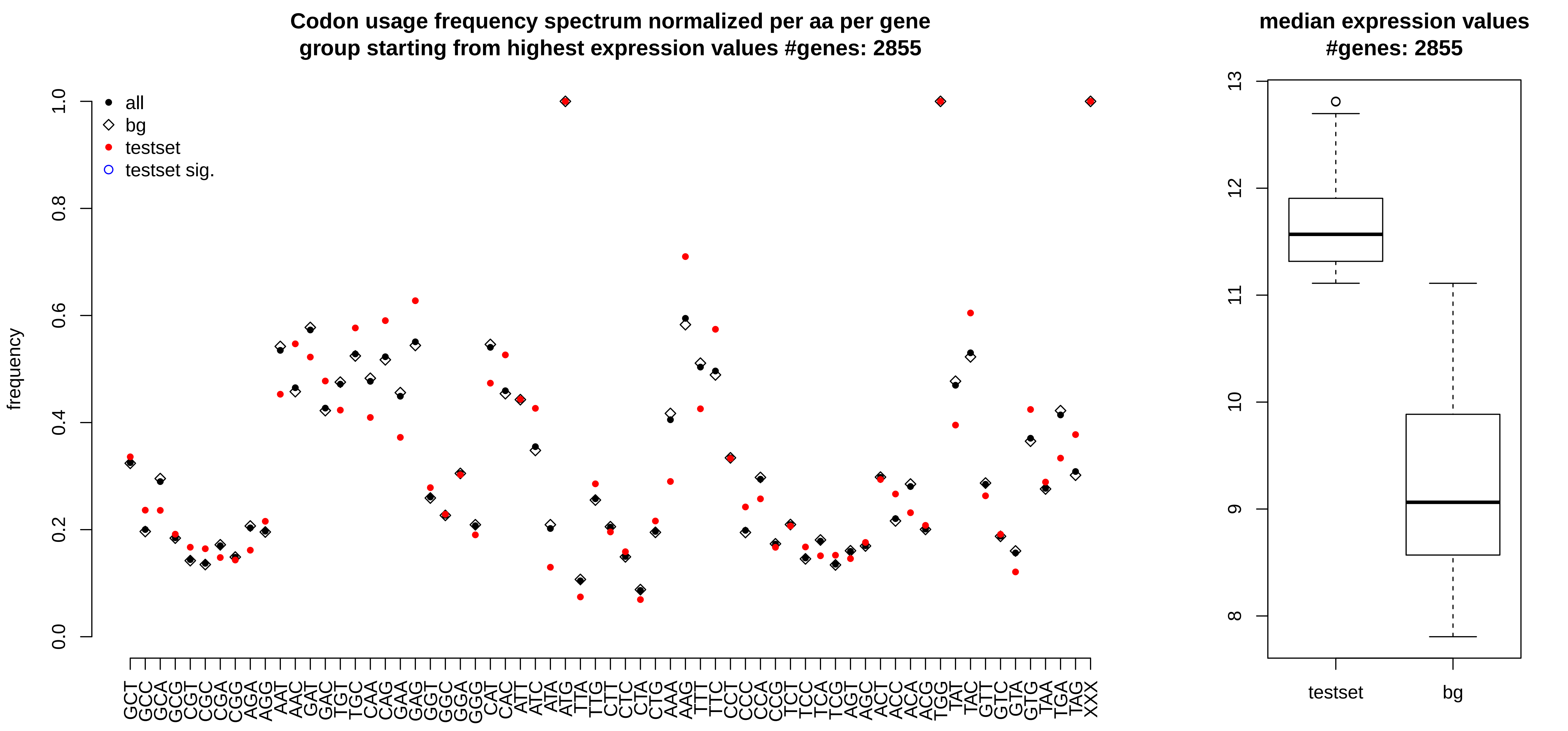

Codon usage frequency spectrum normalized per aa per gene
group starting from highest expression values #genes: 2855
frequency
0.0
0.2
0.4
0.6
0.8
1.0
all
bg
testset
testset sig.
GCT GCC GCA GCG CGT CGC CGA CGG AGA AGG AAT AAC GAT GAC TGT TGC CAA CAG GAA GAG GGT GGC GGA GGG CAT CAC ATT ATC ATA ATG TTA TTG CTT CTC CTA CTG AAA AAG TTT TTC CCT CCC CCA CCG TCT TCC TCA TCG AGT AGC ACT ACC ACA ACG TGG TAT TAC GTT GTC GTA GTG TAA TGA TAG XXX
median expression values
#genes: 2855
8
9
10
11
12
13
testset
bg
